# Sex and age differences in “theory of mind” across 57 countries using the English version of the “Reading the Mind in the Eyes” Test

**DOI:** 10.1073/pnas.2022385119

**Published:** 2022-12-30

**Authors:** David M. Greenberg, Varun Warrier, Ahmad Abu-Akel, Carrie Allison, Krzysztof Z. Gajos, Katharina Reinecke, P. Jason Rentfrow, Marcin A. Radecki, Simon Baron-Cohen

**Affiliations:** ^a^Interdisciplinary Department of Social Sciences, Bar-Ilan University, Ramat Gan 5290002, Israel; ^b^Department of Music, Bar-Ilan University, Ramat Gan 5290002, Israel; ^c^Autism Research Centre, Department of Psychiatry, University of Cambridge, Cambridge CB2 8AH, United Kingdom; ^d^Institut de Psychologie, Université de Lausanne, Lausanne CH-1015, Switzerland; ^e^School of Psychological Sciences, University of Haifa, Haifa 3498838, Israel; ^f^Harvard Paulson School of Engineering and Applied Sciences, Harvard University, Cambridge, MA 02138, United States of America; ^g^Department of Computer Science and Engineering, University of Washington, Seattle, WA 98195-2355, United States of America; ^h^Department of Psychology, University of Cambridge, Cambridge CB2 3EB, United Kingdom; ^i^Social and Affective Neuroscience Group, IMT School for Advanced Studies Lucca, Lucca 55100, Italy

**Keywords:** sex differences, reading the mind in the eyes, cognitive empathy, age differences, cross-cultural

## Abstract

The “Reading the Mind in the Eyes” Test (Eyes Test) is a widely used assessment of “theory of mind.” The NIMH Research Domain Criteria recommends it as one of two tests for “understanding mental states.” Previous studies have demonstrated an on-average female advantage on the Eyes Test. However, it is unknown whether this female advantage exists across the lifespan and across a large number of countries. Thus, we tested sex and age differences using the English version of the Eyes Test in adolescents and adults across 57 countries. We also tested for associations with sociodemographic and cognitive/personality factors. We leveraged one discovery dataset (*N* = 305,726) and three validation datasets (*N*s = 642; 5,284; and 1,087). The results show that: i) there is a replicable on-average female advantage in performance on the Eyes Test; ii) performance increases through adolescence and shallowly declines across adulthood; iii) the on-average female advantage is evident across the lifespan; iv) there is a significant on-average female advantage in 36 out of 57 countries; v) there is a significant on-average female advantage on translated (non-English) versions of the Eyes Test in 12 out of 16 countries, as confirmed by a systematic review; vi) D-scores, or empathizing-systemizing, predict Eyes Test performance above and beyond sex differences; and vii) the female advantage is negatively linked to “prosperity” and “autonomy,” and positively linked to “collectivism,” as confirmed by exploratory country-level analyses. We conclude that the on-average female advantage on the Eyes Test is observed across ages and most countries.

## Significance

In the largest study to date (as far as we know) on the “Reading the Mind in the Eyes” Test (Eyes Test)—a performance task of “theory of mind”—we leveraged four unique datasets (total *N* = 312,739), using the English version of the Eyes Test. We found an on-average female advantage across 57 countries. In line with this is a systematic review of translated (non-English) versions of the Eyes Test identifying an on-average female advantage in eight out of eight different languages. Cross-sectional analyses also showed subtle age differences in Eyes Test scores across the lifespan. We conclude that there is an on-average female advantage across the majority of countries tested. Future research should investigate this in non-English speakers.

“Theory of mind” (ToM) is the ability to attribute mental states to oneself and others, in order to make sense of human behavior and to predict it (1). Since the 1980s, ToM has become central to the study of human development, particularly the development of social perception and social cognition, and to understanding clinical conditions such as autism, conduct disorder, personality disorders, anorexia, and schizophrenia (2–8). ToM is also a central focus of research in comparative psychology, addressing the question of whether ToM is unique to humans (9), to research in neuropsychology, addressing how brain lesions affect ToM (10, 11), and to social neuroscience, testing the biological and social factors that influence ToM (12).

There is evidence that ToM follows consistent developmental patterns during childhood, with a progression through different stages. Although the precursors of ToM in infancy are debated (13), some suggest that precursors of ToM are evident between 9 and 15 mo of age in joint attention behaviors such as gaze following, showing behaviors, and gestures such as pointing to share interest (“protodeclarative pointing”) (14–17). It is notable that autistic children at the earliest point they can be diagnosed show delays or deficits in both joint attention and pretend play, and in later developmental milestones of ToM (1).

In the second year of life, typical children understand the mental states of goals and desires of others (9), and at about 4 y old, children understand that another person can have a different, false belief (the so-called first-order ToM) (18). By around 5 to 6 y of age, children understand what someone is thinking about another person’s mental state (second-order ToM) (19). Later, children also recognize *faux pas*, which is evident around 9–11 y of age and refers to the ability to understand and recognize situations in which someone has said something inappropriate that a listener either did not need to know or which could be hurtful (20). This is relevant to ToM because it is a clear sign that children are monitoring what others know or need to know, and that they have feelings that could be hurt. Autistic children are delayed in passing *faux-pas* tests and autistic adults report finding it hard to justify what is socially appropriate to say or to pick up on when or why someone might have taken offense at what was said (21). ToM abilities continue to develop well in late adolescence (22). Whether developmental progression is identical across countries is debated, with some suggesting its development occurs uniformly across cultures (23), while others suggest it is culture specific (16).

Multiple performance tasks have been developed to measure first-order ToM, including the emotional triangles (24) and as reviewed above, false belief tasks (25). One of the most widely used tasks in the past two decades, particularly in the study of ToM in adults, is the “Reading the Mind in the Eyes” Test (Eyes Test) (26). The Eyes Test is a paper-and-pencil or online performance task where respondents are presented with 36 pictures of the eye region of a human face and asked to indicate which of the four word choices best describes what the person in the picture is thinking or feeling. Reduced performance on the Eyes Test has been reported in autistic individuals (27), those with eating disorders (4), those with personality disorders (7), those with schizophrenia (5), those with substance abuse disorders (28), or those with dementia and Alzheimer’s disease (29). Patients with known brain lesions in the amygdala and inferior frontal gyrus show acquired deficits on the Eyes Test (10, 11). Autistic people and their siblings both show reduced brain activity in these regions during Eyes Test performance in an fMRI scanner (30, 31). These clinical differences suggest that the Eyes Test may be one measure with which to investigate differences in social processes both in individuals with neurodevelopmental and psychiatric conditions and in the general population. Accordingly, The NIMH Research Domain Criteria (RDoC) lists the Eyes Test as one of the two recommended tests for the measurement of individual differences in “understanding mental states” (https://www.nimh.nih.gov/about/advisory-boards-and-groups/namhc/reports/behavioral-​assessment-methods-for-rdoc-constructs.shtml).

There is evidence that both biological and social factors contribute to individual differences in performance on the Eyes Test. In terms of biological factors, performance on the Eyes Test is partly genetic, with a twin heritability of 28% (95% CI: 13 to 42%) and an SNP-based heritability of 5.8% (95% CI: 4.5 to 7.2%) (32). Performance on the Eyes Test is also associated with prenatal testosterone (33), current testosterone (34), and intranasal oxytocin administration (35), implicating biological mechanisms that influence performance on ToM tasks (32, 35–37). In terms of social variables, in adolescents, individual differences in performance on the Eyes Test are associated with smartphone usage, texting, and engaging in fantasy play (38).

Convergence across studies and meta-analyses shows robust sex differences on the Eyes Test, with an on-average female advantage (32, 39). The female advantage could be due to the same set of biological factors that contribute to individual differences in, for example, prenatal testosterone (which is on average higher in males than females) (33), or due to a partly different genetic architecture between males and females (32). In terms of social factors, one potential explanation for the on-average female advantage on the Eyes Test (at least in adolescents and adults) is the gender intensification theory, where the female advantage is seen as partly due to expected gender roles (40). Also relevant is Wood and Eagly’s (41) conceptualization of gender as a biosocial construct that results from complex interactions between biology and experience. It is important to note that an on-average female advantage is not necessarily found across all ToM tasks (42), and some argue that the Eyes Test does not capture ToM but rather emotion recognition (43). Emotion recognition is an important part of ToM, and the Eyes Test captures aspects beyond emotion recognition, as some of the mental states tested include items that are epistemic mental states (such as planning or scheming). When ToM is considered inclusive of emotion recognition, the evidence for the female advantage extends well beyond the Eyes Test, with study samples ranging from 3,000 to 100,000 participants across the lifespan (44–48).

At the geographical level, there are sex differences in personality traits and preferences to study or work in STEM (science, technology, engineering, and mathematics), even in countries that have lower gender inequality (49–53). This so-called gender equality paradox (54) suggests that any residual gender differences in societies with less gender discrimination (e.g., that have moved closer toward gender equality) may reflect partly biological factors. These, in turn, might reflect individual differences that are highly specific (such as greater attention to the eye region of the face, with there being a female advantage in facial recognition (55)) or that are much broader (such as a stronger interest in people than in objects, with there being a greater female social interest (56)). Studying sex is important given that conditions such as autism and schizophrenia, where scores on the Eyes Test are different from the general population, also have a marked sex bias (57). A more comprehensive investigation of the correlates of sex differences on ToM tasks will enable us to better understand the sex differences in conditions such as autism and schizophrenia. There is thus a need for a large-scale, robust study to test these variables definitively.

In contrast to the sex effects on the Eyes Test, age effects on the Eyes Test are less clear. Some studies suggest that scores fluctuate during adolescence, but are stable across adulthood (58). Other studies are contradictory, showing a decrease in scores on the Eyes Test across adulthood (59), which has been replicated with other ToM tasks (60), and others showing an increase in scores across adulthood (61). To our knowledge, there has been no comprehensive investigation of normative age trajectories in performance on the Eyes Test. For instance, it is unknown whether there are large age-related declines in performance on the Eyes Test, and whether there are sex differences in this decline. Although the Eyes Test is widely used, there are gaps in our knowledge, mainly in whether the on-average female advantage generalizes across all countries, and whether there are robust age trends. These gaps in the literature are largely because of a historical reliance on small sample sizes and relatively homogenous samples in terms of both geography and age.

We address these gaps in the literature by using a large and geographically diverse sample to test sex and age differences on the English version of the Eyes Test (the discovery dataset). In addition, we leverage three separate samples (validation datasets A, B, and C) to replicate and extend results from the discovery dataset (validation datasets A and C used the full 36-item version of the Eyes Test and validation dataset B used an 18-item version) (*Methods*). In each of the four studies, participants were asked to indicate their sex, not their gender, although we acknowledge that in common parlance the terms sex and gender are often used interchangeably. We test for: i) on-average sex differences; ii) on-average age differences; iii) associations with demographic variables (including educational attainment), personality/cognitive variables, including the Big Five personality traits (62) and empathizing-systemizing (E-S) cognitive profiles (also referred to as E-S “brain types”) (63). These latter profiles equate to D-scores, the standardized difference between a person’s score on the Empathy Quotient (EQ) (21) and Systemizing Quotient-Revised (SQ-R) (63). E-S brain types have been shown to have a brain basis (64–70). Using country-level data, we further test for: iv) on-average sex-differences across countries; and, as exploratory analyses, v) the association between country-level sociodemographic factors via Political, Economic, Social, and Health (PESH) indicators (71, 72), including the association between gender equality and sex differences on the Eyes Test.

**Fig. 1.**
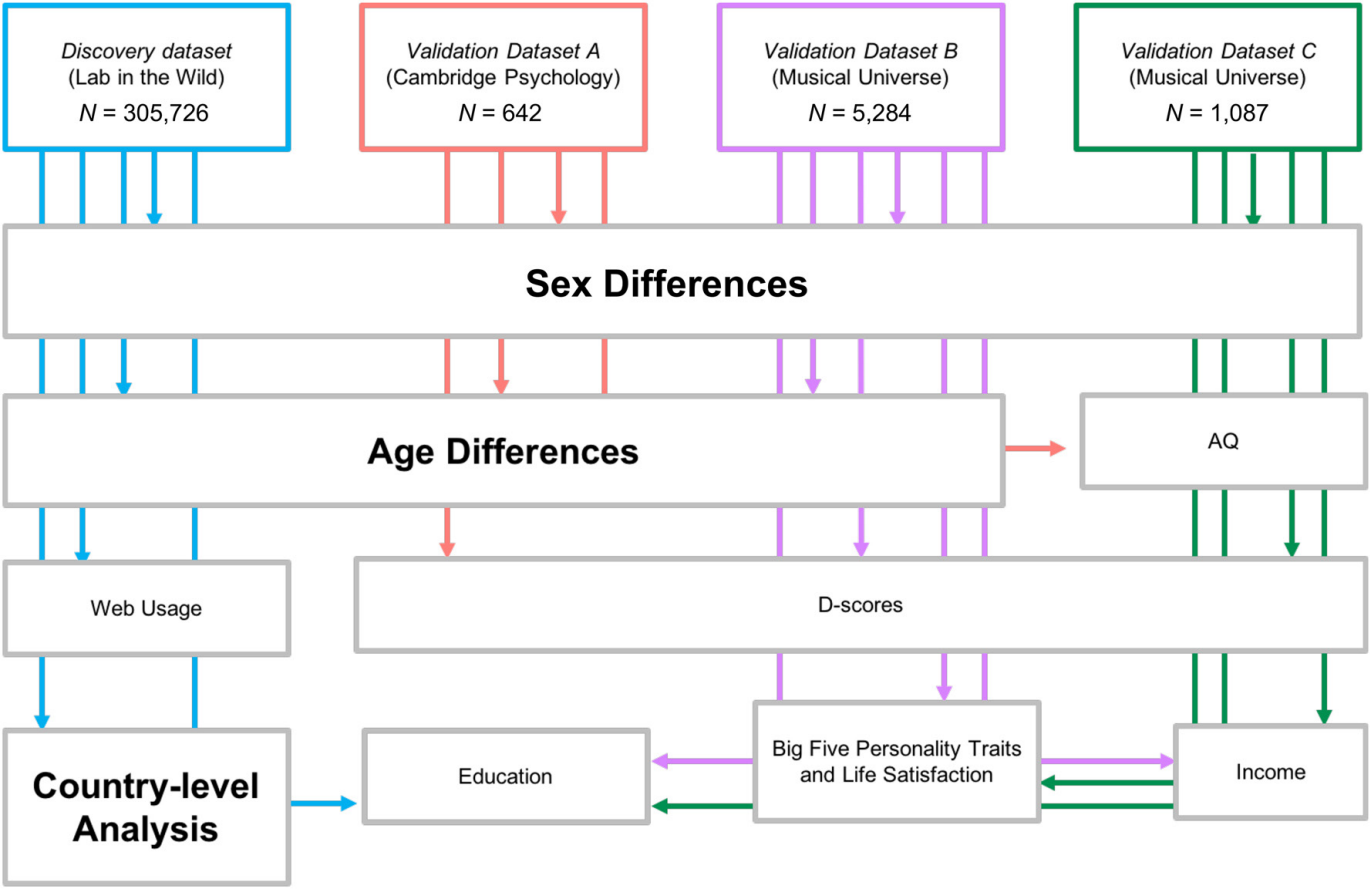
Schematic overview of the study. In this study, we investigated three major questions. Are there on-average sex differences on the Eyes Test; are there on-average age differences on the Eyes Test; and do the on-average sex differences, if any, appear across countries? These three questions are represented by large bold font. We also asked secondary questions: what are the sociodemographic or cognitive/personality factors associated with scores on the Eyes Test and what are the country-level variables (PESH indicators) associated with country-level sex differences on the Eyes Test? Data used to address the former question (including D-scores, Big Five personality traits, education, income) are represented in smaller nonbold font. PESH indicators are not visualized in this diagram. For each question, the primary dataset was the discovery dataset from Lab in the Wild (blue box). We used three validation datasets to validate and extend the results—validation dataset A from Cambridge Psychology (red box), validation dataset B from Musical Universe (purple box), and validation dataset C from Musical Universe (green box). If an arrow appears to go through/underneath a box, then the variable in the box is not included in the specified dataset of the arrow, which can be discerned by the color of the arrow.

The analyses using PESH indicators are exploratory, and while they represent an avenue to understand the possible social mechanisms of the associations, this is not the main aim of our study. Our aim is simply to identify whether sex and age differences are observed across countries. We distinguish country from culture and do not use the terms synonymously here. For instance, India has multiple cultures but is considered a single country/nation. We also conduct a systematic review of studies that used translated versions of the Eyes Test to determine whether sex differences that emerge from our datasets also emerge in non-English speakers and non-English versions of the Eyes Test. Fig. 1 provides a schematic diagram of the study, and sample characteristics are presented in *SI Appendix*, Table S1.

## Results

### Are There On-Average Sex Differences on the Eyes Test?.

**Fig. 2.**
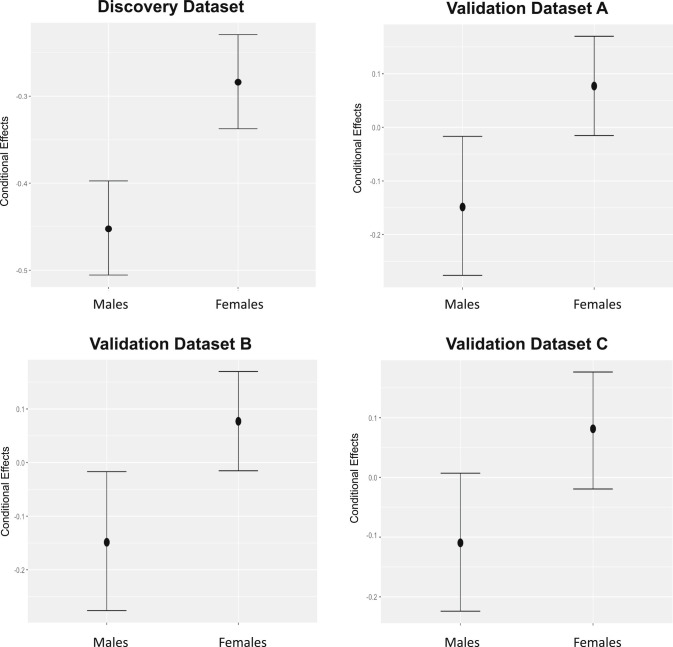
Sex differences on the English version of the Eyes Test across the discovery and validation datasets. Each plot displays the conditional effects of sex (population-level predictor) with 95% credible intervals. As can be seen, there is evidence for an on-average female advantage in each of the four datasets.

For our main analysis, we conducted Bayesian multilevel analysis, using a normal prior: N(0, 1) on the discovery dataset. We estimated sex differences and the prior was applied to Eyes Test scores. Posterior estimates identified an on-average female advantage after including age as a covariate and country as a random intercept (*β* = 0.17; SE = 0.00; 95% CI = [0.16, 0.18]). For each of the three validation datasets, we conducted the same analysis, however, without country as a random intercept since there was not sufficient country-level data in each of the validation samples. Beta estimates equal to 0 provide an estimate of the plausibility that there is no sex difference; beta estimates above 0 indicate a female advantage; and beta estimates below 0 indicate a male advantage. As seen in Table 1, beta estimates are all above 0 and range from 0.17 (SE = 0.00, 95% CI = [0.16, 0.18]) to 0.27 (SE = 0.00, 95% CI = [0.22, 0.32]). As shown in Fig. 2, conditional effects show no overlap in the 95% credible intervals exceeding the 17% overlap threshold for evidential support (73). Taken together, the results shown in Table 1 and Fig. 2 provide robust evidence that females outperformed males across all the four datasets. Reliability on the Eyes Test in each of the four datasets showed acceptable-to-good reliability using McDonald’s Omega total (ω_t_) and ω_h_ (Table 1).

**Table 1.** Sex differences on the Eyes Test in the discovery and validation datasets

			Eyes Test scores		Sex differences			Reliability	
		N	M	SD	Beta	SE	95% CI	ω_t_	ω_h_
Discovery	Females	148,923	27.62	3.92	0.17	0.00	0.16, 0.18	0.80	0.50
	Males	142,694	26.94	4.05					
Validation A	Females	422	26.90	3.50	0.23	0.08	0.07, 0.39	0.76	0.45
	Males	220	26.03	4.32					
Validation B	Females	2,947	14.33	2.02	0.27	0.03	0.22, 0.32	0.74	0.38
	Males	2,293	13.75	2.19					
Validation C	Females	388	28.68	3.33	0.19	0.08	0.03, 0.34	0.75	0.32
	Males	281	28.02	3.23					

This table provides the sample size for each dataset, along with the mean (M) and standard deviation (SD) of Eyes Test scores for each sex, beta, 95% credible intervals, and both ω_t_ and omega hierarchical (ω_h_) reliability coefficients. The maximum possible score on the Eyes Test is 36 for discovery, validation A, and validation C. The total score on the Eyes Test is 18 for validation B.

**Fig. 3.**
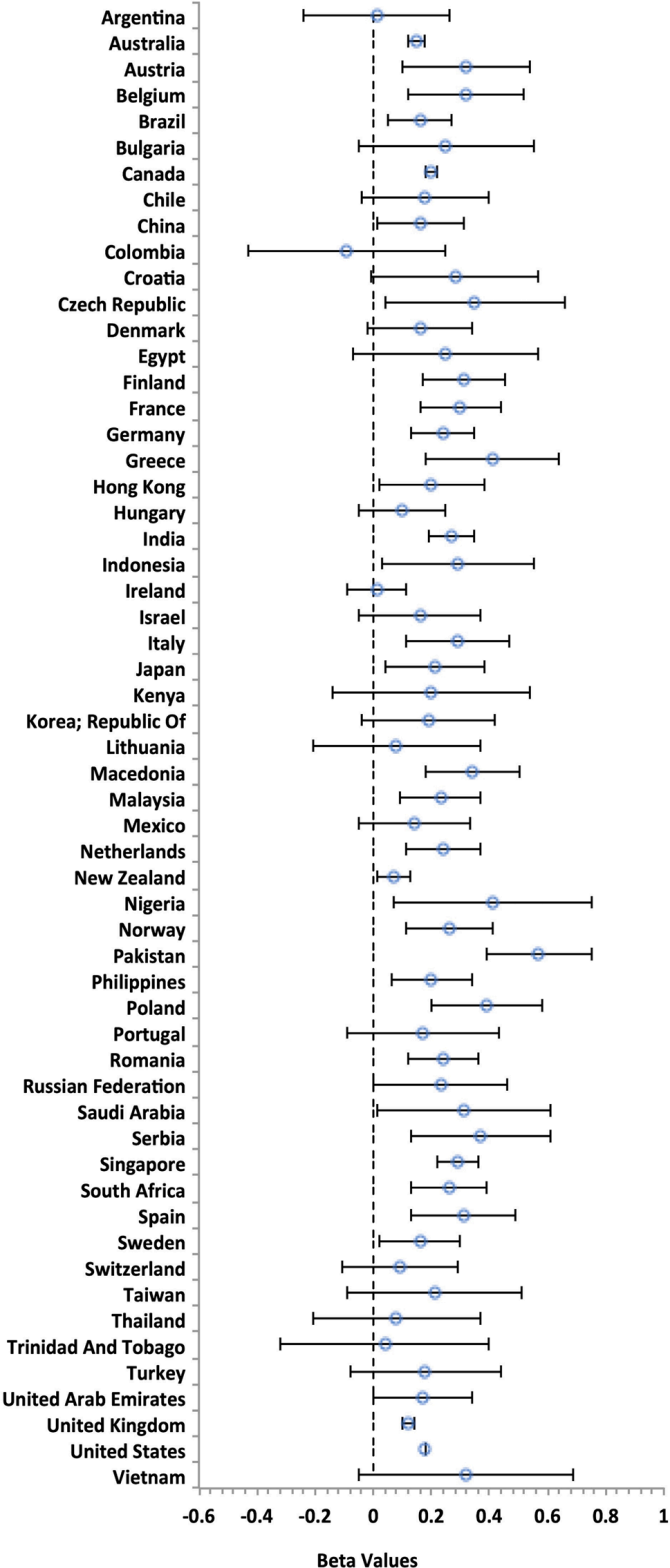
The effect of sex on the English version of the Eyes Test in each of the 57 countries in the discovery dataset. Beta values with 95% credible intervals from multilevel Bayesian analysis are plotted for each of the 57 countries. Beta values above 0 indicate a descriptive female advantage and beta values below 0 indicate a male advantage. As can be seen, 36 countries have a lower interval bound ≥ 0, indicating a female advantage, while no countries have a higher interval bound ≤ 0, which would indicate a male advantage.

With the Bayesian model of the discovery dataset, we examined sex differences within each of the 57 countries that met our inclusion criteria (*Methods*). The sample size per country ranged from *n* = 112 (Vietnam) to *n* = 176,402 (United States) (sample sizes for each country are presented in *SI Appendix*, Table S2). There was only one country in which females did not have a higher descriptive mean score on the Eyes Test than males (i.e., Colombia) (*SI Appendix*, Table S2). As before, beta estimates above 0 indicate a female advantage, while beta estimates below 0 indicate a male advantage. As shown in Fig. 3 and *SI Appendix*, Table S2, analysis of 95% credible intervals showed that there was a female advantage in 36 of the 57 countries (63%). That is, 36 countries had a lower interval bound ≥ 0 providing evidence for a female advantage, while no countries have a higher interval bound ≤ 0, which indicated that no countries had evidence for a male advantage. Facet plots with conditional effects for each sex for each country are presented in *SI Appendix*, Fig S1.

**Fig. 4.**
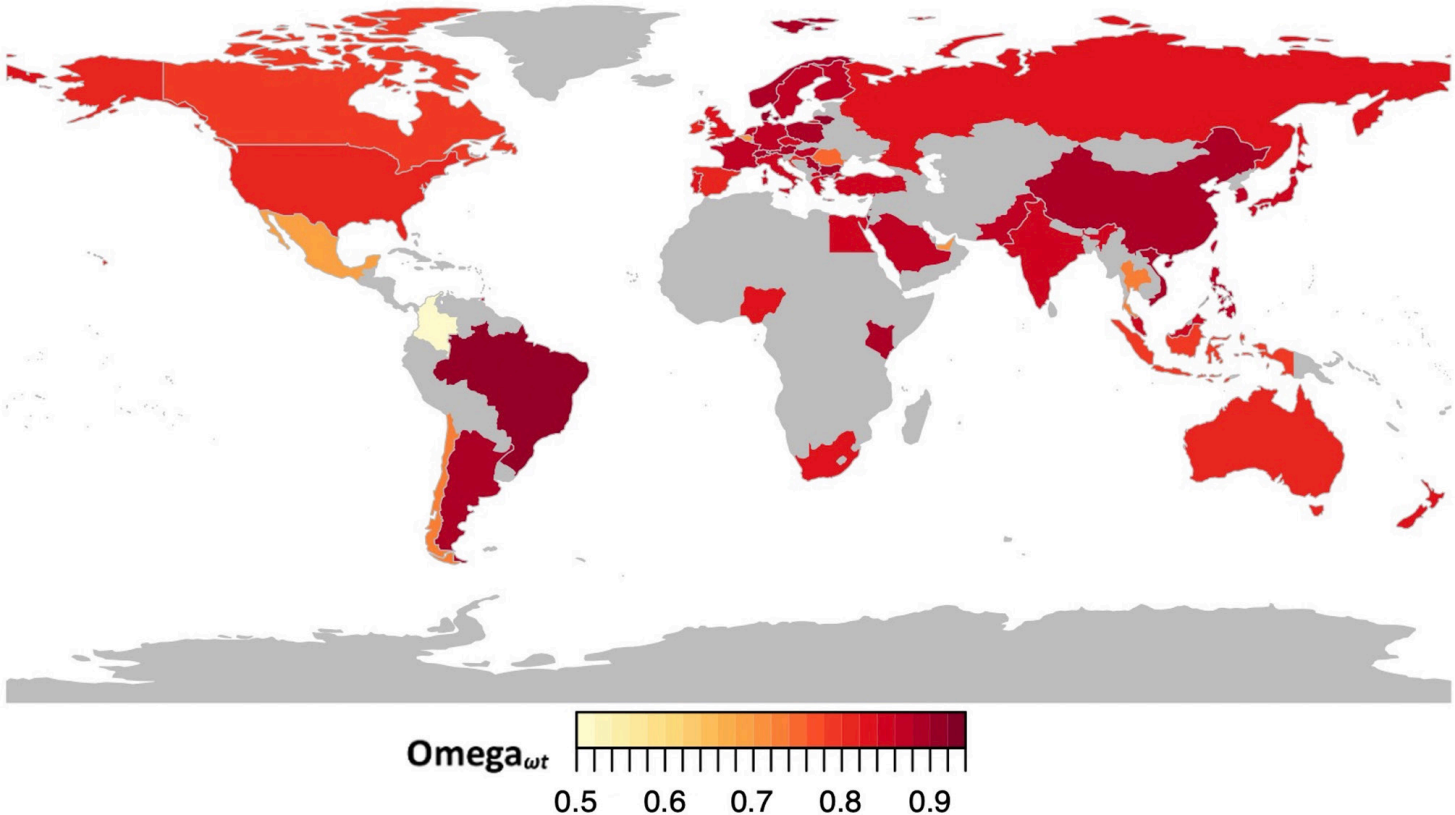
Geographic distribution of ω_t_ for the English version of the Eyes Test across 57 countries. This figure displays ωt for each of the 57 countries observed in the discovery dataset. Lightened yellow colors indicate lower values while darker red colors indicate higher values.

Next, we examined reliability of the Eyes Test within each of the 57 countries in the discovery dataset. As shown in Fig. 4 and *SI Appendix*, Table S2, there was acceptable-to-good reliability for 56 of the 57 countries, with (ω_t_) ranging from 0.68 to 0.92. However, one country, Colombia, had poor reliability with (ω_t_) = 0.49. Since there was a larger sample of countries (*N* = 47) than datasets (*N* = 4), we decided to correlate the beta of each country with the reliability estimates. A high correlation would indicate an effect of reliability on the betas. Column vector correlations from *SI Appendix*, Table S2 between the beta and reliability columns was 0.27, and when first conducting Fisher’s *r* to *z* transformation prior to performing the correlation, it was 0.18, suggesting a minimal effect on the betas.

Our results related to sex differences from the discovery and validation datasets were limited, because we were reliant on proficient speakers of English taking the English version of the Eyes Test. To address this limitation in the data, we conducted a systematic review to identify on-average sex differences in non-English versions of the Eyes Test. There were 16 studies included in the review, with 10 different translations of the Eyes Test. Our study selection process is based on the PRISMA model (*Methods*) and is presented in *SI Appendix*, Fig. S1. Out of the 16 studies selected to review, 12 studies (that include eight translated versions of the Eyes Test) showed a significant female advantage, and the remaining four studies showed a descriptive female advantage that did not reach statistical significance. The study characteristics are presented in *SI Appendix*, Table S3. This shows that an on-average female advantage tends to be found in translated versions of the Eyes Test and validates our findings, which were based on the English version of the Eyes Test.

### Are There On-Average Age Differences on the Eyes Test?.

The large sample in the discovery dataset enabled us to check for on-average sex differences within each age year (i.e., 16, 17, 18, 19, … 70). Our results show that there is indeed a persistent on-average female advantage in each age year (i.e., 16, 17, 18, 19, … 70) (*SI Appendix***,** Table S4). In terms of age itself, results from the Bayesian multilevel model demonstrated that age had a minimal effect within the linear model (*β* = 0.03; SE = 0.00; 95% CI = [0.02, 0.04]) (means, *SD*s, and beta estimates for each age from 16 to 70 are presented in *SI Appendix*, Table S4). To identify peaks (i.e., inflection points) in the age trends, we performed a constrained nonlinear regression analysis separately for females and males within a frequentist model, with a trimmed estimator to obtain a more robust statistic. In terms of age trends for each sex, for females, there were thresholds at 20.25 y of age (*SE* = 0.43; 95% CI = [19.20, 20.69]) and at 49.82 y of age (*SE* = 7.96; 95% CI = [41.18, 63.18]). There was evidence for an increase in performance on the Eyes Test from age 16 to 20.25 y (*β* = 0.40, *SE* = 0.07, 95% CI = [0.26, 0.53]), a shallow decline from age 20.25 to 49.82 y (*β* = −0.013, *SE* = 0.002, 95% CI = [−0.014, −0.008]), and then a further decline—by a factor of 2—from age 49.82 (*β* = −.028, *SE* = 0.047, 95% CI = [−0.159, −0.018]).

**Fig. 5.**
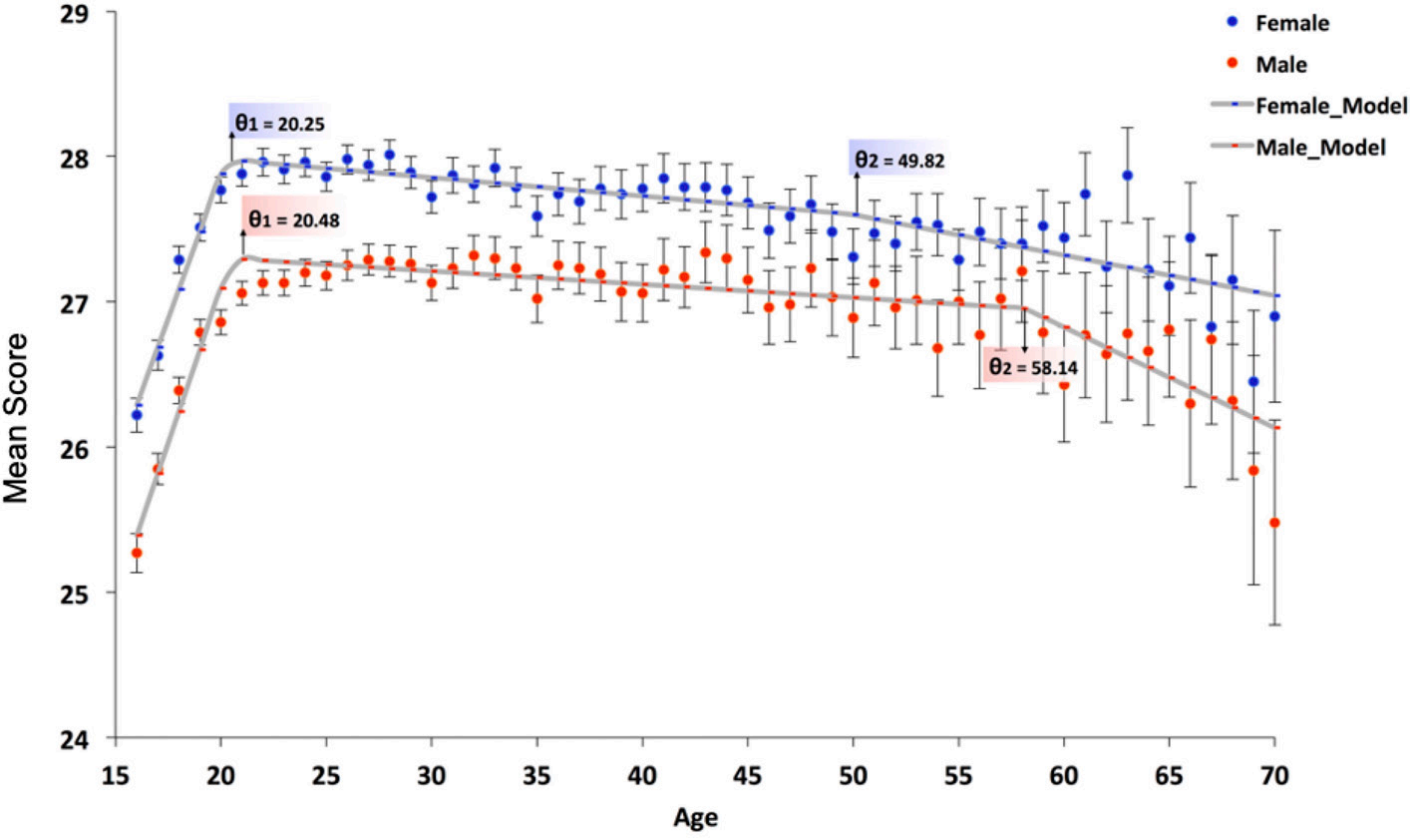
Mean scores on the English version of the Eyes Test by age and sex in the discovery dataset. This figure visualizes the results from the constrained nonlinear regression analysis performed separately for females and males. Results are age and sex differences in Eyes Test scores from 16 to 70 y of age. The figure also identifies inflection points in performance across this age range. Average scores and associated 95% confidence intervals are charted for females and males at each age year.

For males, there were thresholds at 20.48 y of age (*SE* = 0.65; 95% CI = [19.79, 21.58]) and 58.14 y of age (*SE* = 3.36; 95% CI = [56.89, 67.06]), with evidence for an increase from age 16 to age 20.48 (*β* = 0.43, *SE* = 0.08, 95% CI = [0.25, 0.52]), a shallow decline from age 20.48 to age 58.14 (*β* = −0.009, *SE* = 0.002, 95% CI = [−0.012, −0.006]), and then a further steeper decline—by a factor of 8—from age 58.14 (*β* = −0.069, *SE* = 0.134, 95% CI = [−0.486, −0.054]) (Fig. 5). In *SI Appendix*, Fig S3, we provide facet plots for each country, showing age trends from 16 to 70 on the Eyes Test, for females and males separately. These facet plots are based on LOESS regression for each country by sex. Overall, the plots show a similar trend (i.e., Eyes Test scores decline throughout adulthood). Those countries that do not show this trend are countries that have smaller relative sample sizes (e.g., Nigeria).

### Are There Sociodemographic or Cognitive/Personality Associations with Scores on the Eyes Test?.

We expanded our main Bayesian multilevel model by adding sociodemographic and cognitive/personality variables available in each dataset as covariates (*Methods*). First, sex retained an effect on the model in each dataset, even after adding the covariates (*β*s = 0.09 to 0.20) (*SI Appendix*, Table S5). In terms of sociodemographic variables, only education had a positive effect on performance in the discovery (*β =* 0.11, SE = 0.03, 95% CI = [0.04, 0.18]) but not in the validation datasets. There was no evidence that income was a predictor across the datasets. Web usage had a positive effect on the model of the discovery dataset (*β*s *=* 0.15, SE = 0.03, 95% CI = [0.08, 0.22]). In terms of cognitive/personality variables, D-scores (the standardized difference between scores on the EQ and SQ-R), and the basis of “cognitive profile” calculations (high scores indicate a bias toward systemizing and low scores indicate a bias toward empathizing) (*Methods*), had a negative impact on performance in each of the three datasets in which it was included (*β*s *=* −0.13 to −1.06). The Big Five personality traits did not have an effect on performance. Overall, D-scores had the greatest effect on performance, and sex had the second-greatest effect, which underlines the importance of understanding the role of D-scores in performance on the Eyes Test in future research.

### Are On-Average Sex Differences on the Eyes Test Associated with Country-Level Factors?.

Finally, we tested country-level correlates that may shed light on the geographical differences in the magnitude of the female advantage across countries. Since each participant confirmed they understood each word descriptor of each item of the Eyes Test, we conducted an exploratory ecological-correlation analysis at the country level to find associations between on-average sex differences on the Eyes Test and country-level variables. Specifically, we leveraged 16 different country-level variables that outline PESH indicators (71, 72), including gender equality indices (*Methods*). Initial analysis showed that Pakistan was an outlier for the on-average sex differences and Global Gender Gap Index (GGGI) (Mahalanobis distance = 9.00), so we removed it from further analyses.

The 16 PESH indicators were available for 52 of the 56 remaining countries. To reduce the number of indicators, we performed a principal-component analysis (PCA) with varimax rotation. The KMO test for sampling adequacy was 0.72, and there was a clear elbow on the scree plot at four components, suggesting that we retain three components, which together accounted for 86% of the variance. The component loadings are presented in *SI Appendix*, Table S6. Component 1 outlined indicators related to “prosperity” (e.g., Income subindex, Human Development Index, Global Creativity Index), component 2 outlines “autonomy” (e.g., intellectual and affective autonomy, Democracy Index, and egalitarianism), and component 3 outlines “collectivism” (e.g., harmony, and a negative loading for mastery).

We then fitted a Bayesian multilevel model where each of the three components were regressed onto the country-level beta estimate of an Eyes Test score. To account for the fact that the different countries had different sample sizes, we added each country’s sample size as a weight. All the three components demonstrated an effect on the model. Both the prosperity (*β* = −0.15, SE = 0.00, 95% CI = [−0.15, −0.14]) and the autonomy (*β* = −0.11, SE = 0.00, 95% CI = [−0.12, −0.11]) components were negatively associated with beta estimates (*SI Appendix*, Table S7). The collectivism component was positively associated with beta estimates (*β* = 0.08, SE = 0.00, 95% CI = [0.08, 0.09]). This suggests that the more prosperous and autonomous a country is, the smaller the female advantage on the Eyes Test, and the more collectivist a country is, the greater the female advantage.

## Discussion

We confirm an on-average female advantage on the widely used “Reading the Mind in the Eyes” Test (Eyes Test) across four samples assessed with the English version of the test. We show that this on-average female advantage persists across the lifespan from 16 to 70 y of age. Our results also show that the on-average female advantage on the English version of the Eyes Test was evident in 36 of the 57 countries that we observed (in those individuals whose primary or second language was English). There was no country where males scored significantly higher on the Eyes Test than females. Our systematic review of translated versions of the Eyes Test also shows a female advantage that reached statistical significance across 12 of the 16 studies.

Effect sizes can be meaningless without context (74), particularly with complex phenomena like sex differences (75). The effect of sex across the discovery and validation samples could be interpreted as a small or very small effect (76). However, recent theory and research has adopted new benchmarks for interpreting effect sizes which suggest that even small effects can be consequential in the long run (77). Indeed, there is a growing consensus to accept small effects in psychology as the norm and to acknowledge that small effects can have substantial consequences for human behavior. The cumulative evidence from our data that the sex effect appears in multiple countries strengthens the importance for its study. Further research is needed to evaluate how the on-average female advantage on the Eyes Test maps onto real-life outcomes.

We cannot determine causation from our data, as our study does not investigate mechanisms. The robust on-average female advantage on the Eyes Test across countries may have both biological and social determinants. For instance, the gender intensification theory (40) suggests that the observed sex differences might be partly explained by expected gender roles to which children, adolescents, and adults have increased pressure to conform. In terms of biology, there is a significant negative correlation between prenatal testosterone and scores on the Eyes Test (33). Additionally, while SNP heritability for the Eyes Test is identical between the sexes, the genetic correlation between the sexes in adulthood is modest and statistically less than 1 (with 1 being the maximum genetic correlation) (32). This suggests that there may be different genetic pathways underlying the development of ToM between the sexes. In terms of biosocial explanations, it is possible that early sex differences stem from biological factors, but are maintained or amplified by social factors that are prevalent in the countries we observed (78). These potential mechanisms should be investigated in larger studies to better understand what generates these sex differences.

Our findings on age-related trajectories add to prior evidence suggesting age differences in ToM throughout the lifespan (79, 80). For females, our results showed peaks in Eyes Test scores at 20 y of age, with an additional inflection point at 50. For males, there was also a peak at 20 y of age, but an inflection point at 58. The decline in both females and males during later adulthood replicates and extends a previous meta-analysis (*N*s = 790 younger adults and 672 older adults), which showed poorer performance on multiple ToM tasks by older adults compared to younger adults (59). The differences across the lifespan for females and males, coupled with the sex differences on the Eyes Test, raise questions for future research on the role of hormones and their contribution to the development of ToM during adolescents and shallow decline in adulthood.

Our findings also showed that sociodemographic and cognitive/personality factors play a role in performance on the Eyes Test. In particular, D-scores predicted Eyes Test scores above and beyond sex. Similarly, a study of more than 650,000 people found that D-scores accounted for 19 times more of the variance in autistic traits than that of sex and other demographic variables (81). Therefore, evidence is accumulating to show that D-scores play a more important role in different aspects of human cognition than does sex. Separately, the effect of web usage in predicting Eyes Test scores was larger than the effect of sex. While this points to sociocultural factors that influence Eyes Test scores, a likely explanation is that it is simply a reflection of fluency with the computer as a tool.

In our exploratory country-level analyses, we observed that the magnitude of the on-average female advantage in each country had associations with country-level PESH indicators. Specifically, the female advantage on the Eyes Test was positively correlated with cultural values rooted in the collective component and negatively correlated with the prosperity and autonomy components. Prior research shows that individuals with lower social status demonstrate more care and concern for others’ thoughts and feelings (82, 83), attend more closely to the social cues of their partner (84), and are more likely to give financially to others, including giving a higher proportion of their income to charity (85). However, it is unclear how the association between social status and concern for others (86) translates to the on-average female advantage on the Eyes Test. A replication and extension is needed. This points to the added value that country-level analyses bring to our understanding of the Eyes Test and ToM more generally. Taken together, the on-average female advantage on the Eyes Test appears to reduce across more progressive and Westernized countries. These findings lay an initial basis from which future work can build. Rigorous cross-cultural research is needed to shed light on these questions.

Our results provide robust validation for the psychometric properties of the Eyes Test. This is supported by acceptable-to-good reliability of the Eyes Test across datasets and countries. Since the Eyes Test is widely used and is listed as a recommended test for measuring individual differences in “understanding mental states” by the NIMH RDoC, establishing such a validation is useful for researchers intending to use this test. One concern that has been raised about the Eyes Test is that all the 36 stimuli are of white faces. Research findings addressing this concern are mixed. One study found no cultural differences in performance on the Eyes Test when using stimuli of white faces and stimuli of Black faces, regardless of the race of the participant (87). However, some research shows that face processing, more generally, may be biased toward own-race faces (88). Therefore, although studies of the Eyes Test when translated to different languages (89–93) demonstrate that the Eyes Test is a suitable measure for the study of social processes in different geographic contexts, more cross-cultural studies are needed.

Our study had several limitations. First, our study only included English speakers and the English version of the Eyes Test, which limits our conclusions across countries. These English speakers all had access to a computer, suggesting that samples from some countries may be biased and not representative of the demographics of the population. Hence, it is unclear to what extent the on-average sex differences identified in the samples in our study are truly representative of the on-average sex differences in some countries, thereby limiting the interpretation of the cross-country generalizability and of the PESH analyses. Second, while we have endeavored to examine translated versions of the Eyes Test in our systematic review, our study may still be limited in its geographical reach. This may be particularly problematic for countries where English is not spoken widely or with more modest internet and computer access. Additionally, we have not explicitly tested whether performance on the Eyes Test varies between cultures. Future research should explore if the on-average female-advantage is replicated in more traditional societies with minimal exposure to Western culture (94). Third, analysis of age trends was based on cross-sectional rather than longitudinal data, and the age range for the adolescent sample (i.e., ages 16 to 19) was much narrower than for older ages (e.g., 40 to 49). Fourth, ToM was assessed by only a single measure (albeit a widely used and reliable performance measure). Further research is needed to investigate whether other ToM tasks demonstrate a female advantage across ages and countries. Fifth, the adult Eyes Test has not been extensively validated on datasets including participants aged less than 16 y old, and therefore we were unable to test for individual differences in scores in early to middle childhood and early adolescence. Sixth, causation about the observed sex differences in our study cannot be inferred. All these limitations should be addressed in future research.

Although three of the four datasets we used asked participants “what is your sex” rather than “what is your gender,” several of the datasets included answer choices that were nonbinary, including “nonbinary,” “transgender,” and “other.” Furthermore, the question on sex did not specify biological sex assigned at birth. This may have caused confusion among transgender participants and participants who identify as nonbinary. Because of the lack of clarity in the question that was administered, we decided not to include transgender participants in our analysis to test how transgender individuals score on average on the Eyes Test. Furthermore, we do not make any assumptions about how transgender or nonbinary individuals may have responded, and therefore have not speculated about the possible effects of these sampling choices on the results. Our recommendation for future studies is to ask at least two distinct questions to participants: “What is your biological sex assigned at birth?” (with answer choices “female,” “male,” and “intersex”) and “What is your gender identity?” (with answer choices that may include “cisgender,” “transgender,” “agender,” “gender-non-conforming”/“non-binary”/“genderfluid,” “genderqueer,” and “other”).

In conclusion, in one of the largest studies to date on ToM, we found robust evidence in support of an on-average female advantage in ToM using the widely adapted Eyes Test. We were able to replicate the finding in three additional and diverse datasets. The on-average female advantage was present in every age year across the lifespan. We look forward to further research exploring the biological and social determinants of this effect, and how these interact.

## Methods

### Ethics Statement.

Ethical approval for the full study protocol of the discovery dataset was provided by the IRB at Harvard University. Ethical approval for the full study protocol of validation dataset A was provided by the Psychology Research Ethics Committee at the University of Cambridge. Validation datasets B and C were given ethical approval to be used as secondary data by the review board at Ethical and Independent Review Services (http://www.eandireview.com). All participants in each dataset provided informed consent.

## Discovery Dataset.

### Participants and procedures.

More than 460,000 volunteer participants, who were English speaking, completed an English version of the Eyes Test and demographic questions at www.labinthewild.org from February 2013 to May 2019 (95). Participants were asked “If you are not a native speaker of English, did you recognize all the words used to describe emotions?” with four answer choices: 1 = “I am a native speaker of English;” 2 = “I am not a native speaker, but I recognized all the words used to describe emotions in the study;” 3 = “I recognized almost all the words used to describe emotions in the study;” 4 = “I recognized only some of the words used to describe emotions in the study.” For the purposes of this study, we only included participants who indicated either 1 or 2 on the native-English-speaking question. After completing the question items, all participants received feedback about their scores on the Eyes Test.

The Eyes Test has not been fully validated in age groups under 16 y old, as there has only been limited research on the child version of the Eyes Test (96–98), therefore we did not include participants under this age. We did not include people aged above 70 y old, because we lost statistical power above this age group. Including a large age range from 16 to 70 y old enabled us to capture ToM development that is suggested to occur in late adolescence (22). Since the question items across the discovery and validation datasets specified “sex” and not “gender,” we did not include individuals who identified as nonbinary. This left 305,726 individuals for analysis aged 16 to 70 (*M* = 29.57, *SD* = 11.80). 142,696 (48%) were female and the majority of participants were from the United States (*n* = 180,293; 62%) and 30,898 from the United Kingdom (11%). However, because of the large sample size, there was a substantial number of participants from other countries allowing for cross-cultural analysis (*SI Appendix*, Table S2).

### Measures.

**Fig. 6.**
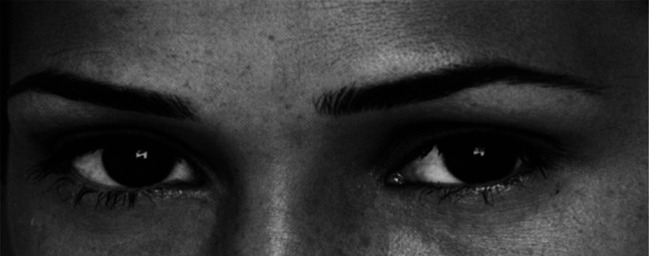
An item from the “Reading the Mind in the Eyes” Test. The photograph in this figure is from item 19 of the Eyes Test. Underneath the photograph are four answer choices: arrogant, grateful, sarcastic, and tentative. The correct answer is tentative.

Participants first completed demographic items and then completed the 36-item Eyes Test (26) (Fig. 6). Demographic items included sex (“What is your gender” with answer choices: 1 = female; 2 = male; and 3 = nonbinary [2019 and after]/it is complicated [prior to 2019]”); age (0 to 123); education (“What is the highest level of education you have received or are pursuing?” with answer choices: 1 = prehigh school; 2 = high school; 3 = college; 4 = masters; 5 = PhD); web usage (“How often do you use a computer?” with answer choices: 1 = once a week or less; 2 = a few times a week; 3 = a couple of hours most days; 4 = many hours on most days); country living in now (“In what country have you spent most of the past 5 y?”); and country lived in during childhood (“In what country did you live most of your childhood? (please pick one that influenced you the most if you grew up in more than one”); and self-reported ability to recognize emotions of others (“Compared to your family and friends, how good are you at reading people’s emotions?” with answer choices: 1 = much worse; 2 = slightly worse; 3 = about the same; 4 = slightly better; and 5 = much better).

## Validation Dataset A.

### Participants and procedures.

Between April 2007 and January 2017, 642 participants completed the Eyes Test at www.cambridgepsychology.com. Of those who indicated, 422 (66%) were female, and the sample ranged in age from 18 to 70 y (*M* = 37.07, *SD* = 12.51). Participants were predominantly from Europe, including 333 (52%) from the United Kingdom. There were no questions about comprehension of the English language in any of the validation datasets and no feedback about scores given to participants. Results on sex differences from a smaller sample (*N* = 320) in this dataset were previously published, and its focus was on sex differences on the Eyes Test in autism (27).

### Measures.

Each measure presented to participants was in English. Participants were asked “What is your sex” with two answer choices: female and male. All participants completed the Eyes Test, and 639 participants also completed the 40-item EQ (21), the 75-item SQ-R, and the 50-item Autism Spectrum Quotient (AQ) (99). The EQ and SQ-R (100) allow for the calculation of D-scores, which is the standardized difference between EQ and SQ-R scores. We followed the procedure established previously for calculating D-scores (63). To calculate the D-score for each participant, we first standardized the EQ and SQ-R scores across the whole sample (including both males and females) based on means from the typical population in the sample: S = [(SQ-R−<SQ-R>)/150 and E = (EQ−<EQ>)/80]. That is, we first subtracted the typical population mean (denoted by <…>) from each individual’s scores, and then divided this by the maximum possible score (150 for the SQ-R, and 80 for the EQ). The D-score is defined as follows: D = S – E. D-scores are often used to provide classifications of five categorical cognitive profiles (sometime referred to in the literature as E-S “brain types”), but since we only used D-scores in linear regressions, we did not specify cognitive profile classifications.

## Validation Dataset B.

### Participants and procedures.

Between March and November 2016, 5,284 participants completed an abbreviated version of the Eyes Test at www.musicaluniverse.org. The participants completed a battery of measures for a larger study on music and personality that involved listening to audio excerpts. The sample was geographically diverse with most of the participants from the United States (*n* = 1,871, 36%) and the United Kingdom (*n* = 793, 15%). Of those who indicated, 2,947 (56%) were female and age ranged from 16 to 70 y (*M* = 33.73, *SD* = 11.76).

### Measures.

As part of the battery, participants completed an 18-item version of the Eyes Test, which included the first half of stimuli of the full Eyes Test. The rationale for administering a brief 18-item version, rather than the full 36-item version, was to prevent participant fatigue. This version is strongly correlated with the full version of the test (*r* = 0.84, *P* < 0.001, *N* = 642 from validation dataset A). Participants were asked “what is your sex” with four answer choices: female, male, transgender, and other. We only included participants in the analysis who selected female or male. Participants completed a brief measure of the Big Five personality traits, the Ten-Item Personality Inventory (101), and the 5-item Satisfaction With Life Scale (102). A subsample of participants also completed the 40-item EQ and the 25-item short version of the SQ (103). D-scores were calculated using the same procedure as in validation dataset A.

## Validation Dataset C.

### Participants, procedures, and measures.

Participants in this dataset were users of the same data collection platform as validation dataset B. However, the only difference was that instead of completing the abbreviated version of the Eyes Test, participants in this sample completed the full version of the Eyes Test. All the remaining measures were the same as those in validation dataset B. There were a total of 1,087 participants. Of those who indicated, 393 (58%) were female, and the sample ranged in age from 16 to 70 y (*M* = 33.98, *SD* = 11.97). Sample characteristics for all the four datasets are presented in *SI Appendix*, Table S1.

### Statistical Analyses.

In the initial analysis, to investigate that the country-wise results are not affected by differing reliabilities of the Eyes Test, we calculated both total McDonald’s Omega (ω_t_, a measure of the total reliability of both the general and the group factors) and hierarchical McDonald’s Omega (ω_h_), a measure of reliability of only the general factor) in all the samples and by country in the discovery dataset.

In the main analysis of sex differences, to accommodate both the individual-level and country-level data in the discovery dataset, we adopted a Bayesian multilevel model that fits all the data, and then showed the posterior distribution of the estimated effect sizes of the sex difference, along with the other parameters being estimated. In the Bayesian multilevel analyses, we specified sex as a fixed variate, age as a covariate, and countries as random intercepts and used a normal prior of (0,1). This was conducted using the *brms* package in R version 4.1.2. For the validation datasets, we also conducted Bayesian multilevel analyses using a normal prior of (0,1), with no random intercepts since the validation datasets did not have enough country-level data.

For analysis of age differences in the discovery dataset, we relied on results from the Bayesian multilevel model above. We also performed constrained nonlinear regression analysis using a frequentist approach (from 16 to 70 y) to identify peaks and inflection points of age trends.

To test for cognitive/personality and sociodemographic variables that are associated with scores on the Eyes Test, we added to the main Bayesian multilevel models in each of the datasets by adding variables that were available in each dataset as covariates: sex, age, education, different country of birth, and web usage in the discovery dataset; sex, age, AQ, and D-scores in the validation A dataset; sex, age, education, income, D-scores, openness, conscientiousness, extraversion, agreeableness, neuroticism, and life satisfaction in the validation B dataset; and sex, education, income, D-scores, openness, conscientiousness, extraversion, agreeableness, neuroticism, and life satisfaction in the validation C dataset.

The large and geographically diverse nature of the discovery dataset gave us the opportunity to test sex differences on the Eyes Test across countries. We determined the participant’s country location based on the country they indicated they are living in now (“In what country have you spent most of the past 5 y?”). Since there are no standards for power analysis in Bayesian modeling to determine the number of countries to retain, we relied on a power analysis using G*Power which suggested a total sample size of *N* = 107 to test for two predictors with an effect size of 0.15 and 95% power. Fifty-seven countries met this criterion.

We tested country-level correlations with sex differences using the PESH framework, which has been previously established and tested successfully in research on geographical psychology (71, 72). For political indicators, we used the EIU Democracy Index from 2018 (https://www.eiu.com/topic/democracy-index) and the IEP Global Peace Index (GPI) from 2019 (http://visionofhumanity.org/app/uploads/2019/06/GPI-2019-web003.pdf). For economic indicators, we used the Income and Education subindices of the United Nations (UN) Human Development Index from 2017 (http://hdr.undp.org/en/data). For social indicators, we used the Global Creativity Index from the Martin Prosperity Institute report in 2015 (http://martinprosperity.org/tag/creativity-index/), the GGGI of the World Economic Forum from 2017 (https://www.weforum.org/reports/the-global-gender-gap-report-2018), the Gender Development Index (GDI) of the UN from 2017 (https://hdr.undp.org/gender-development-index#/indicies/GDI), and Schwartz’s seven culture/society-level value orientations, which are suitable for comparing countries (104) and which were derived from his prior theory on human values (105). Finally, for health indicators, we used the Life Expectancy subindex of the Human Development Index from 2017. We performed a PCA with varimax rotation on the country-level variables and performed a Bayesian multilevel model while specifying the resultant components of the PCA and regressed them onto the country-level beta estimates from sex differences, while adding the sample size of each country as a weight.

To test whether our country-level samples are representative of their countries, we correlated country-level web usage as indicated from self-report in the discovery dataset with the percentage of people in each country who have access to the Internet (gained from Statista and Global Digital Insights) which showed no correlation (*r* = 0.07, *P* = 0.67, *N* = 43). We also correlated the average English comprehension for each country with the 2019 EF English Proficiency Index (https://www.ef.com/wwen/epi/). Here too, there was no significant correlation (*r* = −0.18, *P* = 0.30, *N* = 37). Therefore, our country-level samples may not be representative of the breadth and diversity of each country and the results should be interpreted cautiously and as a basis for generating future hypotheses.

### Systematic Review of Cross-Cultural Studies of the Eyes Test.

Since our data were based on the English version of the Eyes Test, we wanted to observe whether the female advantage was found in translated versions of the Eyes Test. Toward that end, we conducted a systematic review of cross-cultural studies that used translated versions of the Eyes Test. We used the PRISMA model to identify, screen, establish eligibility, and include studies in the review (106). We searched for studies that met the following criteria: 1) published from 2001 (upon first publication of the Eyes Test) until February 2021; 2) included the adult version (36 items) of the Eyes Test (not the child version) that was not modified in any way except for translations into another language (e.g., not shortened, lengthened, or with altered photograph stimuli); 3) included nonclinical samples; 4) analyzed sex effects in their sample, including those that did find sex differences and those that did not; 5) *n* > 20 for each sex; and 6) was published in English. We first searched PubMed for the terms: “Reading the Mind in the Eyes,” “Eyes Test,” and “RMET.” We then searched Google Scholar for the same terms, and added an additional search term, “translate/d,” to identify additional studies that administered a translated version of the Eyes Test. The literature search was conducted from January 2021 to March 2021. We excluded studies that were only abstracts, conference proceedings, or gray literature. From each study, we extracted the study meta data, reported means, standard deviations, and results from significant testing for each study that met the inclusion criteria. The resulted PRISMA diagram is presented in *SI Appendix*, Fig. S1.

## Supplementary Material

Appendix 01 (PDF)

## Data Availability

Scripts used to analyze the data have been deposited in the Open Science Framework, https://osf.io/hu63x/?view_only=ce5703afb2aa4529bdb0def3beafdc15. Some data may become available (because participants were not asked to consent for their data, even anonymized, to be made publicly available, it is only available on request via a Visitor Agreement with the University of Cambridge, if appropriate, and under the existing ethical approval).
