# Supplementary material for: Sex and age differences in “theory of mind” across 57 countries using the English version of the “Reading the Mind in the Eyes” Test
Source: Proc Natl Acad Sci U S A. 2022 Dec 30;120(1):e2022385119. doi: 10.1073/pnas.2022385119 (PMC9910622; doi:10.1073/pnas.2022385119)
Supplement: Supplementary file 1 — Appendix 01 (PDF) [file pnas.2022385119.sapp.pdf]

Supplemental Information Appendix

Table S1. Sample characteristics for discovery and validation datasets.

	Discovery dataset	Validation dataset A	Validation dataset B	Validation dataset C
Data collection platform	Lab in the Wild	Cambridge Psychology	Musical Universe	Musical Universe
N	305,726	642	5,284	1,087
% Female	48%	66%	56%	56%
Age range	16 to 70	18 to 70	13 to 70	16 to 70
M Age	29.57	37.07	33.73	33.98
SD Age	11.8	12.51	11.76	11.87
%US	62%	32%	36%	29%
%UK	11%	52%	15%	24%
Tests	Eyes Test	Eyes Test, EQ, SQ-R	Eyes Test, EQ, SQ-25, TIPI	Eyes Test-18, EQ, SQ-25, TIPI

Table S2. On average sex differences on the Eyes Test across 57 countries. The table provides the sample sizes (N), mean scores (M), standard deviations (SD), beta and 95% CIs for 57 countries from the discovery dataset.

Country	Omega	Females			Males			Sex differences		
		N	M	SD	N	Mean	SD	Beta	SE	95% CI
Argentina	.87	95	25.24	6.2	158	25.19	4.68	0.01	0.13	-.24, .26
Australia	.79	7,533	27.86	3.84	7,895	27.29	3.95	0.15	0.02	.12, .18
Austria	.87	133	26.63	5.5	156	24.83	5.54	0.32	0.11	.09, .54
Belgium	.69	171	27.73	3.62	246	26.46	3.98	0.32	0.1	.13, .52
Brazil	.89	572	25.54	4.44	708	24.84	4.48	0.16	0.06	.05, .27
Bulgaria	.87	77	25.86	4.61	86	24.67	4.6	0.25	0.16	-.07, .55
Canada	.78	10,433	27.8	3.71	10,619	27.05	3.9	0.2	0.01	.17, .22
Chile	.71	138	25.84	3.71	208	25.18	3.69	0.18	0.11	-.02, .40
China	.87	344	26.67	3.93	351	26	4.55	0.16	0.08	.01, .31
Colombia	.49	62	25.65	3.9	79	25.99	3.61	-0.09	0.27	-.43, .25
Croatia	.80	80	26.85	3.79	96	25.64	4.48	0.28	0.15	.00, .57
Czech Republic	.84	56	27.05	4.72	106	25.49	4.05	0.35	0.16	.04, .66
Denmark	.90	186	27.05	4.51	318	26.29	5	0.16	0.09	-.02, .34
Egypt	.83	96	27.04	3.76	65	25.88	5.26	0.25	0.16	-.06, .57
Finland	.86	367	27.83	4.26	469	26.48	4.29	0.31	0.07	.18, .45
France	.85	390	27.36	4.2	503	26.07	4.11	0.3	0.07	.18, .44
Germany	.85	658	27.23	4.16	845	26.16	4.52	0.24	0.05	.14, .35
Greece	.84	131	26.93	3.94	150	25.13	4.43	0.41	0.12	.18, .64
Hong Kong	.75	239	27.55	3.76	216	26.74	4.38	0.2	0.09	.02, .38
Hungary	.85	324	23.71	4.77	347	23.21	5.05	0.1	0.08	-.05, .25
India	.83	1,074	26.22	4.38	1,353	24.99	4.7	0.27	0.04	.19, .35
Indonesia	.77	125	24.94	4.25	110	23.51	5.53	0.29	0.13	.03, .55
Ireland	.80	748	27.55	4.01	1,002	27.51	3.71	0.01	0.05	-.09, .11
Israel	.92	168	27.08	3.83	194	26.42	4.19	0.16	0.11	.05, .37
Italy	.84	228	26.86	4.31	226	25.52	4.71	0.29	0.09	.10, .47
Japan	.84	240	27.34	3.73	328	26.47	4.49	0.21	0.08	.04, .38
Kenya	.87	61	25.54	4.54	62	24.55	4.81	0.2	0.18	-.15, .54
Korea; Republic Of	.86	136	27.68	3.93	182	26.84	4.65	0.19	0.11	-.02, .42
Lithuania	.90	91	23.6	6.65	95	23.07	6.21	0.08	0.15	-.22, .37
Macedonia	.83	403	21.05	4.95	233	19.25	5.7	0.34	0.08	.17, .50
Malaysia	.83	401	26.69	4.26	310	25.69	4.32	0.23	0.07	.08, .37
Mexico	.68	155	25.93	4.41	224	25.34	3.96	0.14	0.1	-.06, .33
Netherlands	.85	410	27.62	3.84	549	26.6	4.46	0.24	0.06	.12, .37

New Zealand	.82	2,032	27.61	3.86	1,838	27.35	3.73	0.07	0.03	.01, .13
Nigeria	.82	62	25.34	4.44	69	23.29	5.04	0.41	0.17	.08, .75
Norway	.88	263	27.16	4.19	455	25.99	4.63	0.26	0.08	.11, .41
Pakistan	.85	250	25.4	4.93	183	22.13	6.12	0.57	0.09	.38, .75
Philippines	.85	505	25.99	4.34	296	25.1	4.82	0.2	0.07	.05, .34
Poland	.87	174	26.67	4.52	233	24.76	5	0.39	0.1	.19, .58
Portugal	.82	96	26.77	4	163	26.08	3.85	0.17	0.13	-.19, .43
Romania	.74	498	25.99	4.76	530	24.79	5.29	0.24	0.06	.12, .36
Russian Federation	.81	128	26.99	4.23	162	25.99	4.53	0.23	0.11	.00, .46
Saudi Arabia	.86	96	26.46	5.28	72	24.81	4.89	0.31	0.16	.01, .61
Serbia	.87	89	26.22	4.78	120	24.27	5.33	0.37	0.14	.10, .61
Singapore	.73	1,811	27.56	3.47	1,470	26.5	3.68	0.29	0.03	.23, .36
South Africa	.81	488	28.04	3.89	489	26.98	3.98	0.26	0.06	.14, .39
Spain	.80	242	27.21	3.73	244	25.89	4.62	0.31	0.09	.13, .49
Sweden	.86	257	27.01	4.46	549	26.28	4.52	0.16	0.07	.02, .30
Switzerland	.88	176	27.05	4.97	199	26.65	4.1	0.09	0.1	-.12, .29
Taiwan	.85	75	26.99	3.77	90	26.12	4.26	0.21	0.16	-.10, .51
Thailand	.72	78	26.76	4.08	100	26.43	4.19	0.07	0.15	-.22, .37
Trinidad And Tobago	.85	78	26.42	4.57	59	26.22	4.54	0.05	0.18	-.30, .40
Turkey	.83	117	27	4.78	124	26.19	4.36	0.18	0.13	-.08, .44
United Arab Emirates	.70	291	26.88	3.88	220	26.24	3.82	0.17	0.09	-.01, .34
United Kingdom	.82	14,005	27.58	3.97	16,268	27.08	4.17	0.12	0.01	.10, .14
United States	.79	93,259	27.79	3.66	83,143	27.13	3.83	0.17	0	.17, .18
Vietnam	.88	58	26.6	3.98	54	25.19	4.67	0.32	0.18	-.05, .69

Table S3. Study characteristics for studies included in the systematic review

study citation	language	included (no/yes)	significant female advantage	significant male advantage	nonsignificant sex difference	descriptive but nonsignificant female advantage
Tollenaar MS, Overgaauw S. Empathy and mentalizing abilities in relation to psychosocial stress in healthy adult men and women. Heliyon. 2020 Aug 14;6(8):e04488. doi: 10.1016/j.heliyon.2020.e04488. PMID: 32904299; PMCID: PMC7452492.	Dutch	yes	no	no	yes	yes
Schmitt HS, Sindermann C, Li M, Ma Y, Kendrick KM, Becker B, Montag C. The Dark Side of Emotion Recognition - Evidence From Cross-Cultural Research in Germany and China. Front Psychol. 2020 Jul 9;11:1132. doi: 10.3389/fpsyg.2020.01132. PMID: 32733302; PMCID: PMC7363803.	German	yes (sample 2)	yes	no	no	no
Kynast J, Polyakova M, Quinque EM, Hinz A, Villringer A, Schroeter ML. Age- and Sex-Specific Standard Scores for the Reading the Mind in the Eyes Test. Front Aging Neurosci. 2021 Jan 28;12:607107. doi: 10.3389/fnagi.2020.607107. PMID: 33633559; PMCID: PMC7902000.	German	yes	yes	no	no	no
Kynast J, Quinque EM, Polyakova M, Luck T, Riedel-Heller SG, Baron-Cohen S, Hinz A, Witte AV, Sacher J, Villringer A, Schroeter ML. Mindreading From the Eyes Declines With Aging - Evidence From 1,603 Subjects. Front Aging Neurosci. 2020 Oct 22;12:550416. doi: 10.3389/fnagi.2020.550416. PMID: 33192452; PMCID: PMC7656776.	German	yes	no	no	yes	yes
Voracek, M., & Dressler, S. G. (2006). Lack of correlation between digit ratio (2D: 4D) and Baron-Cohen's "Reading the Mind in the Eyes" test, empathy, systemising, and autism-spectrum quotients in a general population sample. *Personality and Individual Differences*, *41*(8), 1481-1491.	German	yes	yes	no	no	no
Vellante M, Baron-Cohen S, Melis M, Marrone M, Petretto DR, Masala C, Preti A. The "Reading the Mind in the Eyes" test: systematic review of psychometric properties and a validation study in Italy. Cogn Neuropsychiatry. 2013 Jul;18(4):326-54. doi: 10.1080/13546805.2012.721728. Epub 2012 Oct 30. PMID: 23106125; PMCID: PMC6345369.	Italian	yes	yes	no	no	no

Preti A, Vellante M, Petretto DR. The psychometric properties of the "Reading the Mind in the Eyes" Test: an item response theory (IRT) analysis. Cogn Neuropsychiatry. 2017 May;22(3):233-253. doi: 10.1080/13546805.2017.1300091. Epub 2017 Mar 13. PMID: 28288549.	Italian	yes	no	no	yes	yes
Lee HR, Nam G, Hur JW. Development and validation of the Korean version of the Reading the Mind in the Eyes Test. PLoS One. 2020 Aug 31;15(8):e0238309. doi: 10.1371/journal.pone.0238309. PMID: 32866184; PMCID: PMC7458289.	Korean	yes	no	no	yes	yes
Schmitt HS, Sindermann C, Li M, Ma Y, Kendrick KM, Becker B, Montag C. The Dark Side of Emotion Recognition - Evidence From Cross-Cultural Research in Germany and China. Front Psychol. 2020 Jul 9;11:1132. doi: 10.3389/fpsyg.2020.01132. PMID: 32733302; PMCID: PMC7363803.	Mandarin Chinese	yes (sample 1)	yes	no	no	no
Khorashad BS, Baron-Cohen S, Roshan GM, Kazemian M, Khazai L, Aghili Z, Talaei A, Afkhamizadeh M. The "Reading the Mind in the Eyes" Test: Investigation of Psychometric Properties and Test-Retest Reliability of the Persian Version. J Autism Dev Disord. 2015 Sep;45(9):2651-66. doi: 10.1007/s10803-015-2427-4. PMID: 25832800.	Persian	yes	yes	no	no	no
Jankowiak-Siuda, K., Baron-Cohen, S., Bialaszek, W., Dopierala, A., Kozlowska, A., & Rymarczyk, K. (2016). Psychometric Evaluation Of The'reading The Mind In The Eyes'test With Samples Of Different Ages From A Polish Population. *Studia Psychologica*, *58*(1), 18.	Portuguese	yes	yes	no	no	no
Megías-Robles A, Gutiérrez-Cobo MJ, Cabello R, Gómez-Leal R, Baron-Cohen S, Fernández-Berrocal P. The 'Reading the mind in the Eyes' test and emotional intelligence. R Soc Open Sci. 2020 Sep 16;7(9):201305. doi: 10.1098/rsos.201305. PMID: 33047068; PMCID: PMC7540806.	Spanish	yes	yes	no	no	no
Redondo I, Herrero-Fernández D. Validation of the Reading the Mind in the Eyes Test in a healthy Spanish sample and women with anorexia nervosa. Cogn Neuropsychiatry. 2018 Jul;23(4):201-217. doi: 10.1080/13546805.2018.1461618. Epub 2018 Apr 11. PMID: 29635964.	Spanish	yes	yes	no	no	no
Hallerbäck MU, Lugnegård T, Hjärthag F, Gillberg C. The Reading the Mind in the Eyes Test: test-retest reliability of a Swedish version. Cogn Neuropsychiatry. 2009 Mar;14(2):127-43. doi: 10.1080/13546800902901518. PMID: 19370436.	Swedish	yes	yes	no	no	no

Yıldırım EA, Kaşar M, Güdük M, Ateş E, Küçükparlak I, Ozalmete EO. Investigation of the reliability of the "reading the mind in the eyes test" in a Turkish population. Turk Psikiyatri Derg. 2011 Fall;22(3):177-86. English, Turkish. PMID: 21870307.	Turkish	yes	yes	no	no	no

Table S4. *N*s, Means, *SD*s, and sex differences on the Eyes Test for each age

Age	Females					Males					Sex difference		
	N	M	SD	95% CI		N	M	SD	95% CI		*B*	SE	95% CI
16	5097	26.22	4.24	26.10	26.34	4289	25.27	4.52	25.14	25.41	.22	.02	.19, .25
17	6456	26.63	4.23	26.53	26.73	6238	25.85	4.37	25.74	25.96	.18	.02	.15, .21
18	6917	27.29	3.93	27.19	27.38	8327	26.39	4.22	26.29	26.48	.22	.02	.19, .25
19	6587	27.51	3.89	27.42	27.61	8011	26.79	4.04	26.70	26.88	.18	.02	.15, .21
20	7073	27.77	3.78	27.68	27.85	8790	26.86	4.08	26.78	26.95	.23	.02	.20, .26
21	7465	27.88	3.74	27.80	27.96	9247	27.06	3.98	26.98	27.14	.21	.02	.18, .24
22	6408	27.96	3.84	27.87	28.06	8041	27.13	3.92	27.05	27.22	.21	.02	.18, .25
23	5806	27.91	3.82	27.82	28.01	7408	27.13	3.89	27.04	27.22	.20	.02	.17, .24
24	5621	27.96	3.72	27.86	28.06	6810	27.20	3.93	27.11	27.29	.20	.02	.15, .23
25	5466	27.86	3.77	27.76	27.96	6520	27.18	4.02	27.08	27.27	.17	.02	.14, .21
26	5101	27.98	3.65	27.88	28.08	5313	27.25	3.89	27.15	27.36	.19	.02	.15, .23
27	4906	27.94	3.76	27.83	28.04	5049	27.29	3.81	27.18	27.39	.17	.02	.13, .21
28	4655	28.01	3.64	27.91	28.12	4676	27.28	3.78	27.17	27.39	.20	.02	.16, .24
29	4384	27.89	3.71	27.78	28.00	4035	27.26	3.89	27.14	27.38	.17	.02	.13, .21
30	4509	27.72	3.83	27.61	27.83	4086	27.13	3.94	27.01	27.25	.15	.02	.11, .19
31	3556	27.87	3.68	27.75	27.99	3234	27.23	4.02	27.09	27.37	.17	.02	.12, .21
32	3426	27.81	3.72	27.69	27.94	3124	27.32	3.88	27.18	27.45	.13	.03	.08, .18
33	3218	27.92	3.73	27.79	28.05	2654	27.30	3.85	27.15	27.44	.16	.03	.11, .22
34	2956	27.79	3.69	27.65	27.92	2508	27.23	3.78	27.08	27.38	.15	.03	.10, .20
35	2874	27.59	3.80	27.45	27.73	2322	27.02	4.00	26.85	27.18	.15	.03	.09, .20
36	2459	27.74	3.75	27.59	27.89	1988	27.25	3.76	27.08	27.41	.13	.03	.07, .19
37	2320	27.69	3.78	27.53	27.84	1954	27.23	3.96	27.06	27.41	.12	.03	.06, .18
38	2249	27.78	3.67	27.62	27.93	1710	27.19	3.93	27.01	27.38	.15	.03	.09, .21
39	1956	27.74	3.80	27.57	27.91	1474	27.07	3.97	26.87	27.27	.17	.04	.10, .24
40	2149	27.78	3.79	27.62	27.94	1604	27.06	4.05	26.86	27.26	.18	.03	.12, .25
41	1751	27.85	3.60	27.68	28.02	1273	27.22	3.89	27.01	27.44	.17	.04	.10, .24
42	1934	27.79	3.62	27.63	27.95	1356	27.17	3.96	26.96	27.38	.17	.03	.10, .23
43	1867	27.79	3.73	27.63	27.96	1301	27.34	3.89	27.13	27.55	.12	.04	.05, .19
44	1699	27.77	3.74	27.59	27.95	1148	27.30	3.96	27.07	27.53	.12	.04	.05, .20
45	1739	27.68	3.82	27.50	27.86	1266	27.15	4.12	26.93	27.38	.13	.04	.06, .20
46	1528	27.49	3.79	27.30	27.68	1026	26.96	4.13	26.71	27.21	.13	.04	.06, .21
47	1503	27.59	3.68	27.41	27.78	925	26.98	3.99	26.72	27.24	.16	.04	.08, .24
48	1406	27.67	3.77	27.47	27.86	887	27.23	4.04	26.96	27.49	.11	.04	.03, .20
49	1413	27.48	3.70	27.28	27.67	815	27.03	3.85	26.77	27.30	.12	.04	.03, .21
50	1651	27.31	3.94	27.12	27.50	969	26.89	4.34	26.61	27.16	.10	.04	.02, .18

51	1201	27.47	4.01	27.24	27.70	668	27.13	3.88	26.84	27.43	.08	.05	-.01, .18
52	1325	27.40	3.52	27.21	27.59	725	26.96	3.91	26.68	27.25	.12	.05	.03, .21
53	1327	27.55	3.63	27.35	27.74	676	27.01	4.03	26.71	27.32	.14	.05	.05, .24
54	1159	27.53	3.71	27.32	27.75	588	26.68	4.09	26.35	27.01	.22	.05	.12, .32
55	1258	27.29	3.79	27.08	27.50	657	27.00	3.82	26.71	27.30	.08	.05	-.02, .17
56	1067	27.48	3.84	27.25	27.71	505	26.77	4.21	26.40	27.14	.18	.05	.07, .28
57	951	27.40	3.84	27.16	27.65	486	27.02	3.98	26.67	27.38	.10	.06	-.01, .21
58	860	27.40	3.79	27.15	27.66	463	27.21	3.86	26.86	27.56	.05	.06	-.06, .16
59	835	27.52	3.65	27.28	27.77	378	26.79	4.18	26.37	27.21	.19	.06	.07, .31
60	957	27.44	3.87	27.19	27.68	438	26.43	4.22	26.03	26.83	.25	.06	.14, .36
61	624	27.74	3.65	27.46	28.03	320	26.77	3.94	26.34	27.20	.26	.07	.13, .39
62	616	27.24	4.00	26.92	27.55	312	26.64	4.23	26.17	27.12	.14	.07	.01, .28
63	496	27.87	3.72	27.54	28.20	265	26.78	3.80	26.32	27.25	.28	.08	.13, .43
64	473	27.22	3.91	26.86	27.57	245	26.66	4.07	26.15	27.17	.14	.08	-.02, .29
65	493	27.11	3.87	26.77	27.45	284	26.81	4.01	26.34	27.28	.08	.08	-.07, .22
66	359	27.44	3.68	27.06	27.82	202	26.30	4.17	25.72	26.88	.29	.09	.12, .46
67	282	26.83	4.15	26.34	27.31	170	26.74	3.89	26.15	27.32	.02	.10	-.17, .21
68	249	27.15	3.57	26.71	27.60	162	26.32	3.52	25.77	26.87	.23	.10	.03, .43
69	206	26.45	3.60	25.96	26.95	129	25.84	4.57	25.04	26.63	.15	.11	-.07, .37
70	192	26.90	4.18	26.31	27.50	128	25.48	4.08	24.76	26.19	.34	.11	.11, .56

Table S5. Results from multi-level regressions where sex and age, and sociodemographic and cognitive/personality variables predict Eyes Test scores within each dataset

**Discovery dataset**							
	Beta	SE	95% CI		Rhat	Bulk ESS	Tail ESS
Intercept	-.98	.24	-1.46	-.52	1.00	1985	2508
sex (0 = male; 1 = female)	.14	.04	.06	.23	1.00	6102	3090
age	.00	.00	.00	.01	1.00	5177	2599
education	.11	.03	.04	.18	1.00	7078	2980
web usage	.15	.03	.08	.22	1.00	7336	2536
comprehension	-.33	.09	-.50	-.16	1.00	1473	1994
face knowledge	.05	.02	.00	.09	1.00	6813	3098
**Validation dataset A**							
	Beta	SE	95% CI		Rhat	Bulk ESS	Tail ESS
Intercept	.17	.17	-.15	.51	1.00	4613	2847
sex (0 = male; 1 = female)	.20	.09	.03	.38	1.00	4565	2787
age	.00	.00	-.01	.00	1.00	5722	2753
autism quotient	-.01	.01	-.02	.00	1.00	3038	3102
D score	-.13	.21	-.54	.29	1.00	3248	3290
**Validation dataset B**							
	Beta	SE	95% CI		Rhat	Bulk ESS	Tail ESS
Intercept	.01	.19	-.36	.38	1.00	3925	3356
sex (0 = male; 1 = female)	.16	.04	.09	.24	1.00	4063	3160
age	.00	.00	.00	.01	1.00	5043	3368
education	.00	.02	-.04	.03	1.00	4620	2853
income	.00	.00	-.01	.01	1.00	6709	2524
D score	-1.06	.12	-1.29	-.83	1.00	3810	3221
openness	-.01	.02	-.04	.02	1.00	4474	3273
conscientiousness	.00	.01	-.03	.03	1.00	4349	3279
extraversion	-.02	.01	-.04	.00	1.00	3941	3067
agreeableness	-.01	.02	-.04	.03	1.00	3897	3043
neuroticism	-.01	.01	-.03	.01	1.00	4232	3539
life satisfaction	.01	.01	-.02	.04	1.00	4370	3249
**Validation dataset C**							
	Beta	SE	95% CI		Rhat	Bulk ESS	Tail ESS
Intercept	-.45	.54	-1.50	.60	1.00	4101	3268
sex (0 = male; 1 = female)	.09	.11	-.13	.30	1.00	5402	3020
age	.00	.01	-.01	.01	1.00	5546	3126
education	.02	.05	-.08	.12	1.00	6040	3358

income	.00	.01	-.03	.02	1.00	5050	2886
D score	-.74	.33	-1.39	-.13	1.00	4321	3105
openness	.01	.05	-.09	.12	1.00	5603	2806
conscientiousness	.02	.04	-.06	.10	1.00	4652	3417
extraversion	.03	.04	-.04	.09	1.00	4881	3011
agreeableness	.06	.05	-.03	.15	1.00	4863	3508
neuroticism	-.03	.04	-.10	.04	1.00	5164	3452
life satisfaction	-.01	.04	-.09	.08	1.00	4341	3147

Table S6. Results from PCA analyses at the country-level

	Component		
	Prosperity	Autonomy	Collectivism
income_index	0.95	0.12	0.10
human_development_index	0.95	0.27	0.06
ci_globalcreativityindex	0.90	0.26	-0.06
global_peace_index	-0.90	-0.23	0.11
education_index	0.89	0.39	0.02
human_inequality_coefficient	-0.85	-0.40	0.00
gender_development_index	0.78	0.25	0.26
schwartz_embedded	-0.25	-0.93	-0.09
schwartz_intel_autonomy	0.05	0.90	0.30
schwartz_aff_autonomy	0.34	0.82	-0.30
schwartz_hierarchy	-0.32	-0.81	-0.28
GGGI	0.49	0.73	-0.10
democracy_index	0.47	0.68	-0.30
schwartz_egalitarianism	0.30	0.68	0.37
schwartz_mastery	-0.32	0.08	-0.88
schwartz_harmony	-0.28	0.32	0.82

This table shows component loadings from PCA analysis with varimax rotation at the country-level.

Table S7. Bayesian multi-level analysis at the country-level to show associations between three country-level indices and the country-level female advantage on the Eyes Test.

	Beta	SE	95% CI		Rhat	Bulk ESS	Tail ESS
Intercept	-0.3	0	-0.31	-0.29	1	3956	2973
component_1_prosperity	-0.15	0	-0.15	-0.14	1	3640	2872
component_2_autonomous	-0.11	0	-0.12	-0.11	1	4419	2905
component_3_collectivism	0.08	0	0.08	0.09	1	2619	2761

This table reports results from Bayesian multi-level analysis at the country-level predicting beta weights from sex-differences, with country-sample size added as a weight.

Fig. S1. Facet plots showing sex differences on the Eyes Test in each of 57 countries in the discovery dataset.

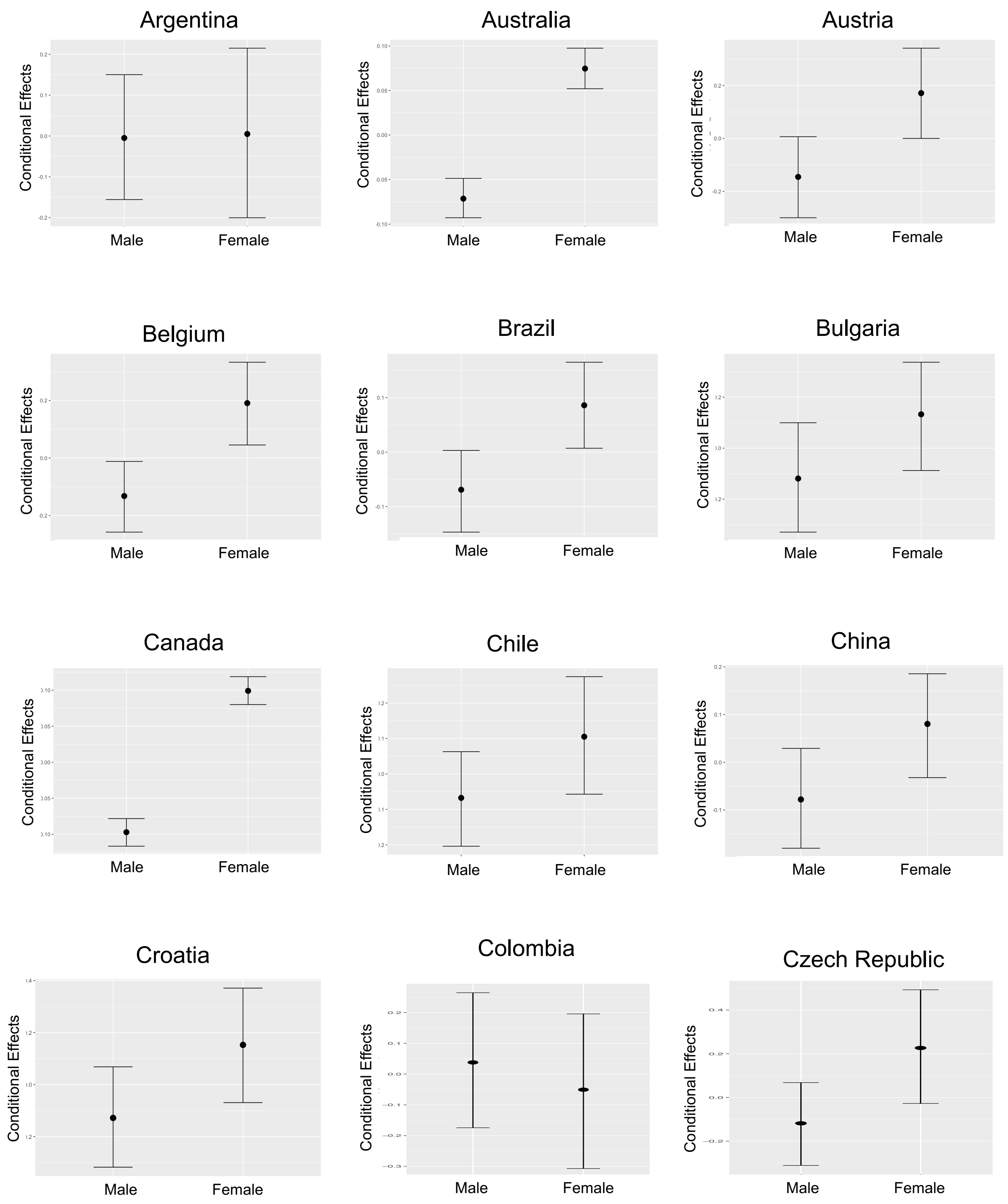

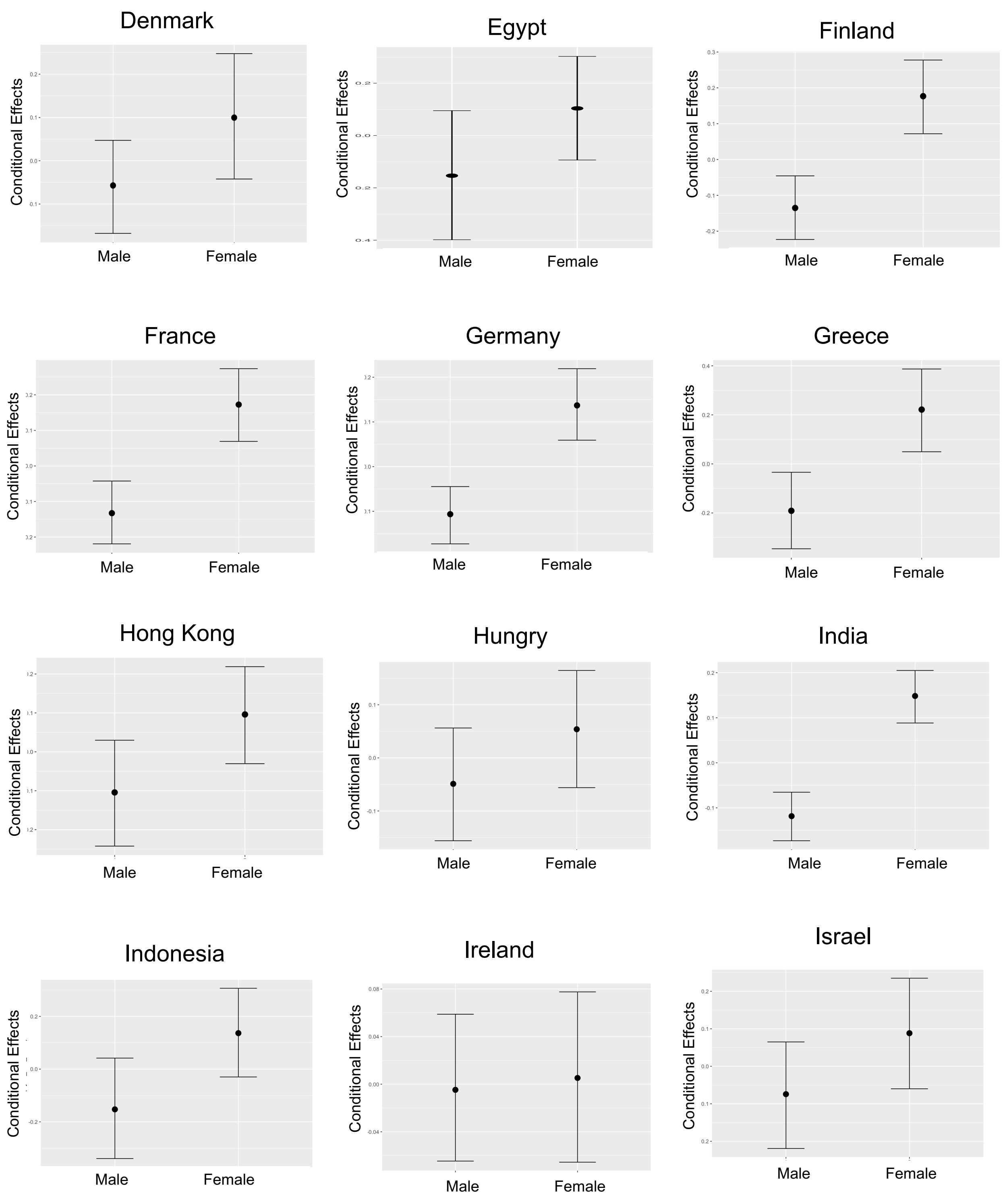
Denmark
Egypt
Finland
France
Germany
Greece
Hong Kong
Hungry
India
Indonesia
Ireland
Israel
Conditional Effects
Male
Female

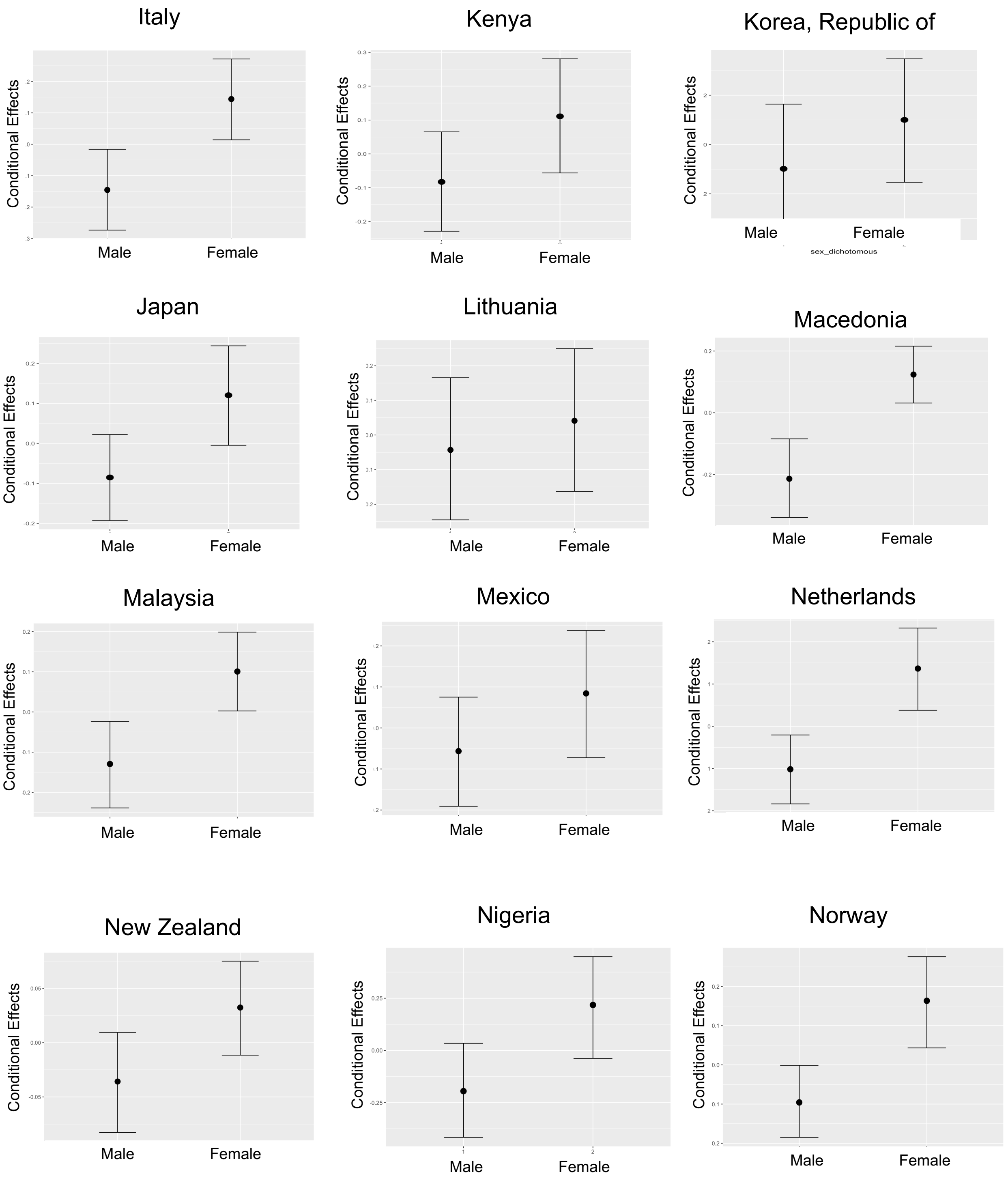
Italy
Conditional Effects
Male
Female
Kenya
Conditional Effects
Male
Female
Korea, Republic of
Conditional Effects
Male
Female
sex_dichotomous
Japan
Conditional Effects
Male
Female
Lithuania
Conditional Effects
Male
Female
Macedonia
Conditional Effects
Male
Female
Malaysia
Conditional Effects
Male
Female
Mexico
Conditional Effects
Male
Female
Netherlands
Conditional Effects
Male
Female
New Zealand
Conditional Effects
Male
Female
Nigeria
Conditional Effects
Male
Female
Norway
Conditional Effects
Male
Female

Pakistan

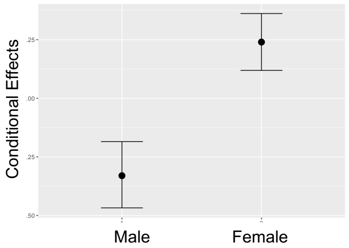

Philippines

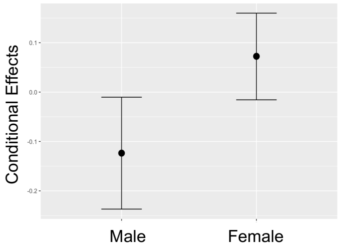

Poland

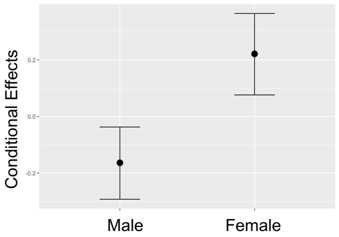

Portugal

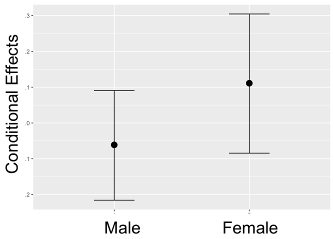

Romania

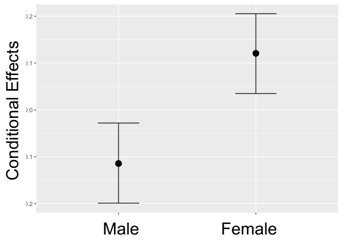

Russian Federation

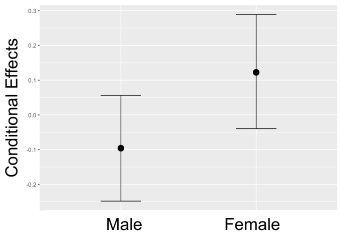

Saudi Arabia

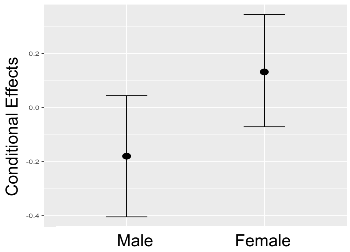

Serbia

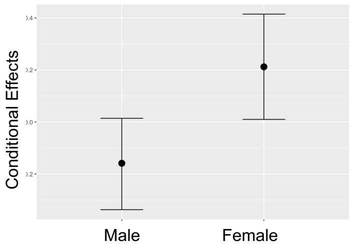

Singapore

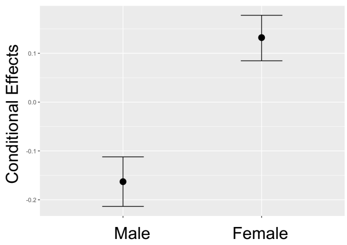

South Africa

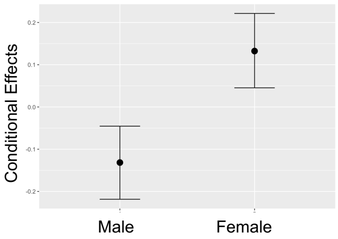

Spain

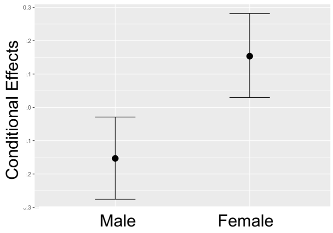

Sweden

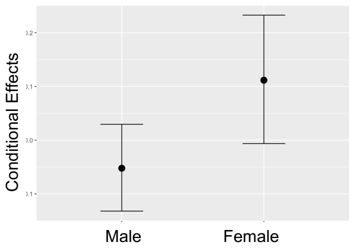

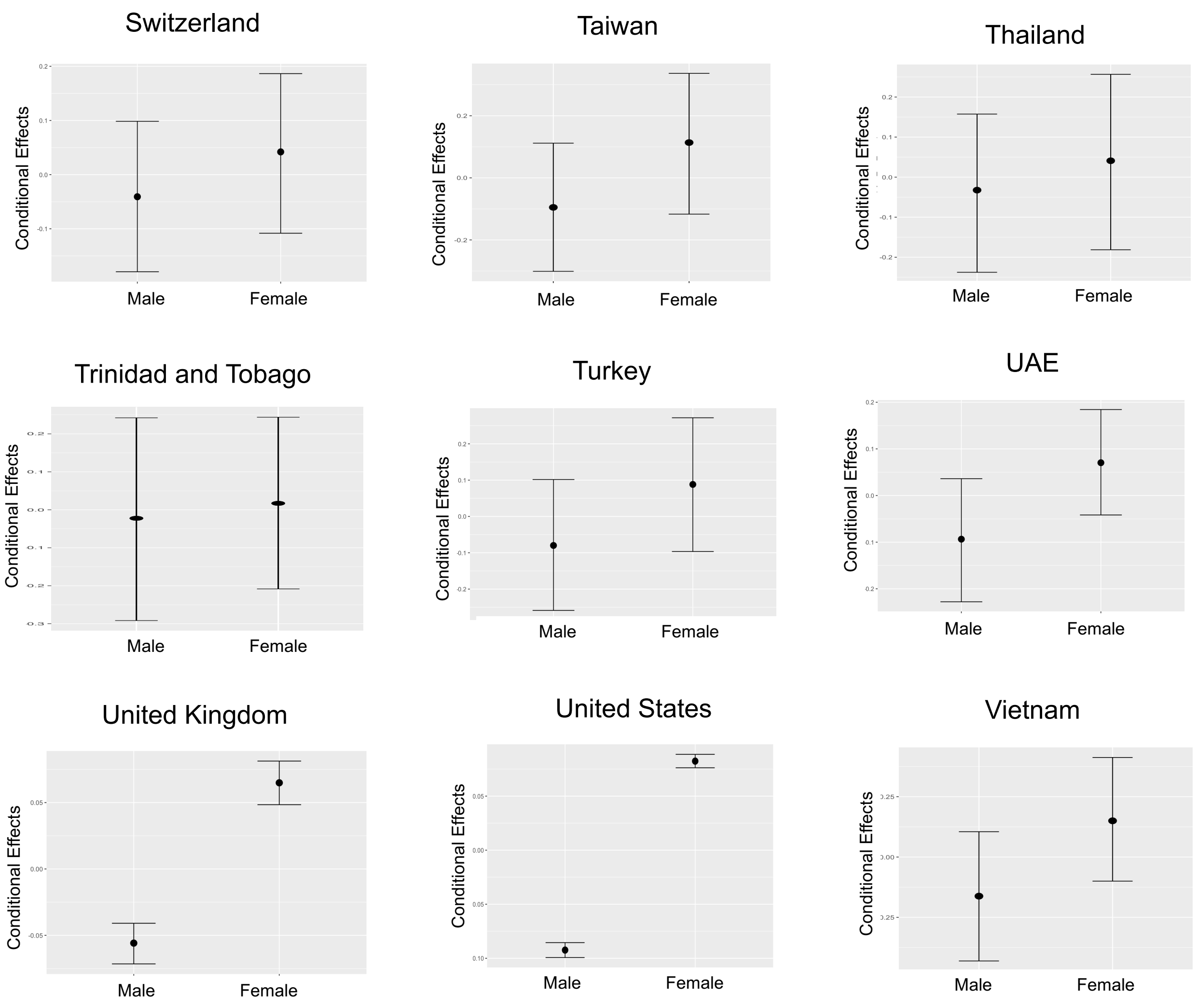

This figure provides facet plots for each country based on sex differences on the Eyes Test. The x-axis in each of the plots is sex (1 = male (dots/bars on the left side of each graph); 2 = female (dots/bars on the right side of each graph). The y-axis on each plot is beta estimates from Bayesian regression models where sex predicts Eyes Test scores.

Fig. S2. Selection Process for the Systematic Review based on the PRISMA model.

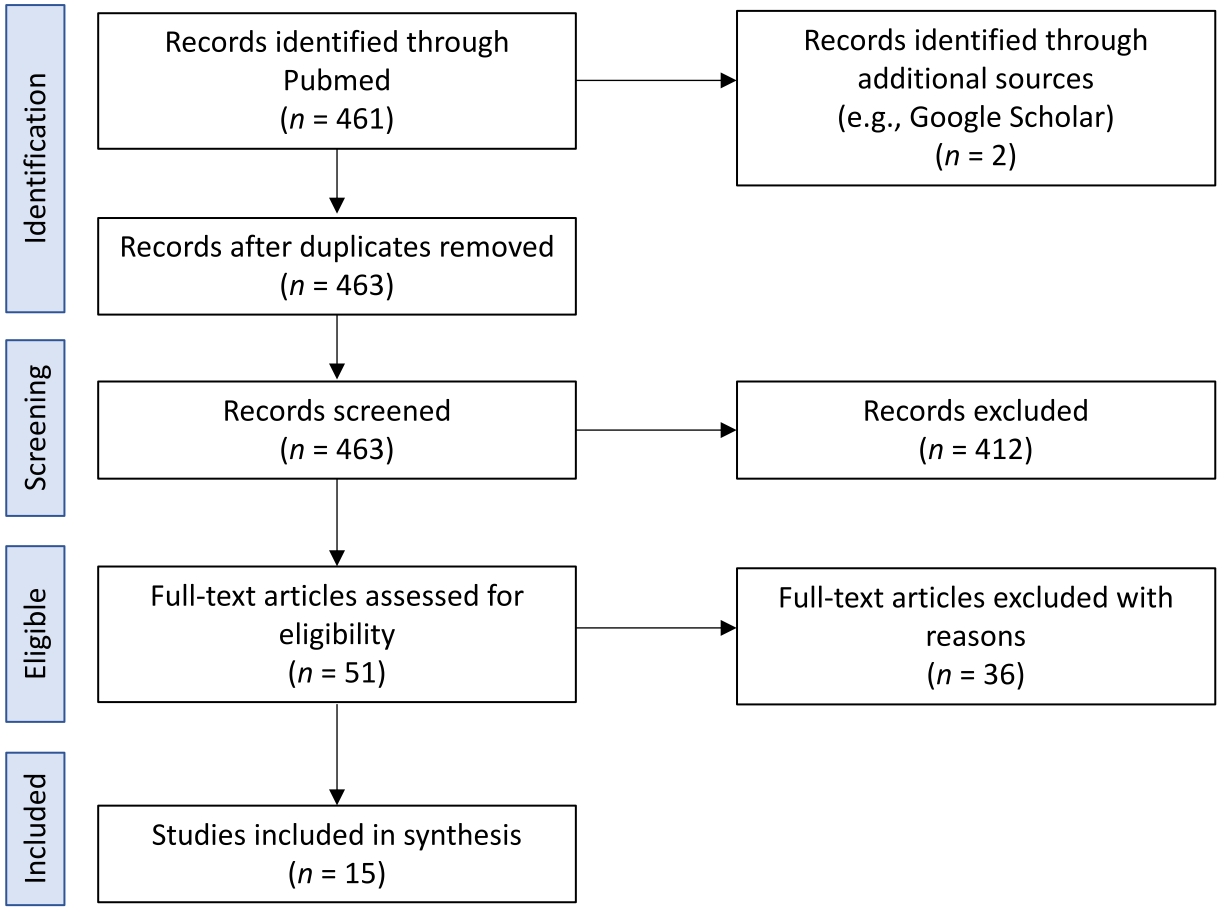

This figure presents the step-by-step selection process of the systematic review based on the PRISMA model. We searched for studies that met the following criteria: (1) published from 2001 (upon first publication of the Eyes Test) until February 2021; (2) Included the adult version of the Eyes Test (not the child version); (3) Included non-clinical samples; and (5) Reported sex differences in their sample; (3) n > 20 for each sex.

Fig. S3. Facet plots showing age trends on the Eyes Test per country, separated by sex, in the discovery dataset

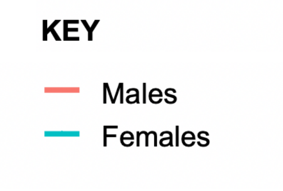

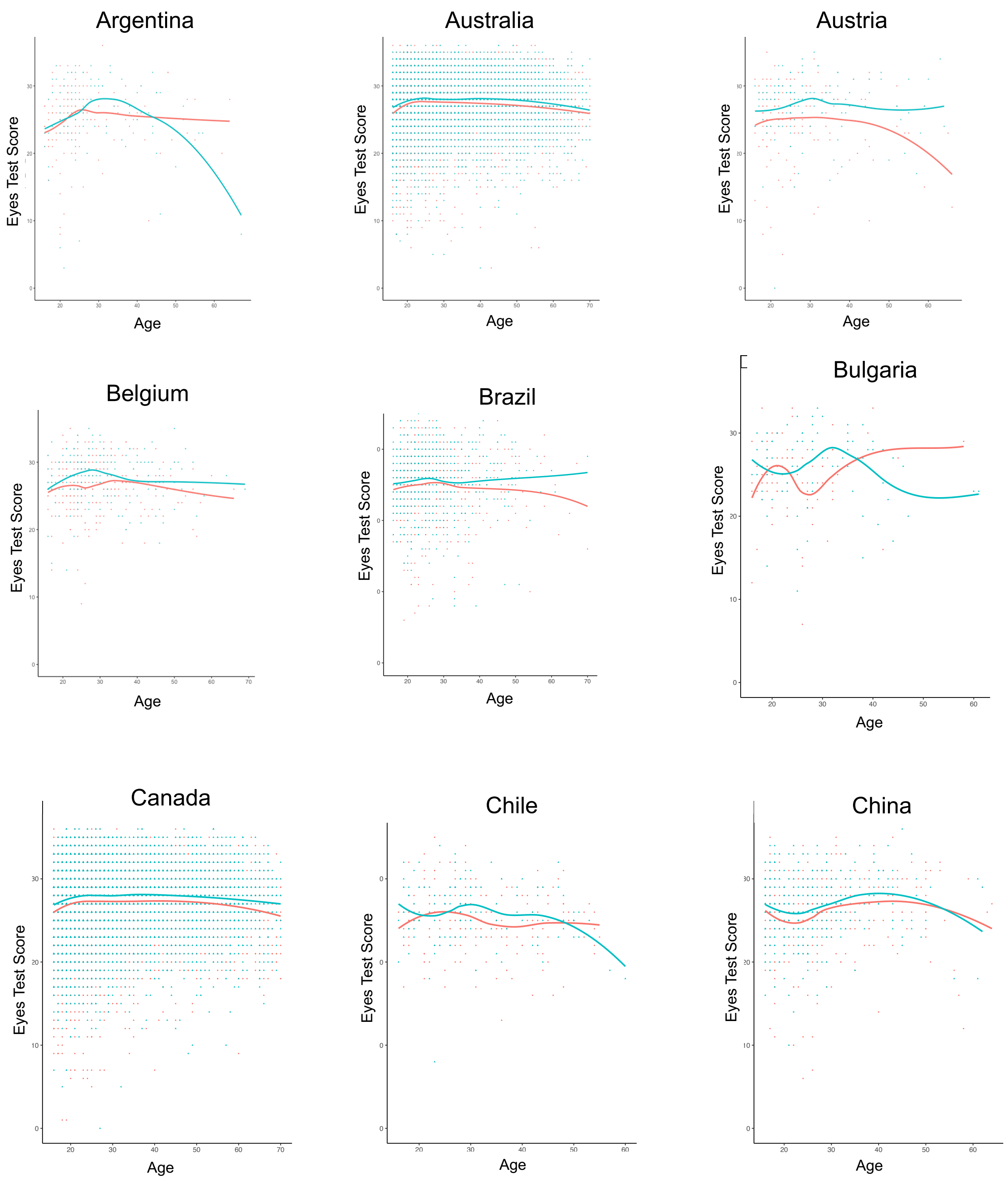
Argentina
Australia
Austria
Belgium
Brazil
Bulgaria
Canada
Chile
China
Eyes Test Score
Age

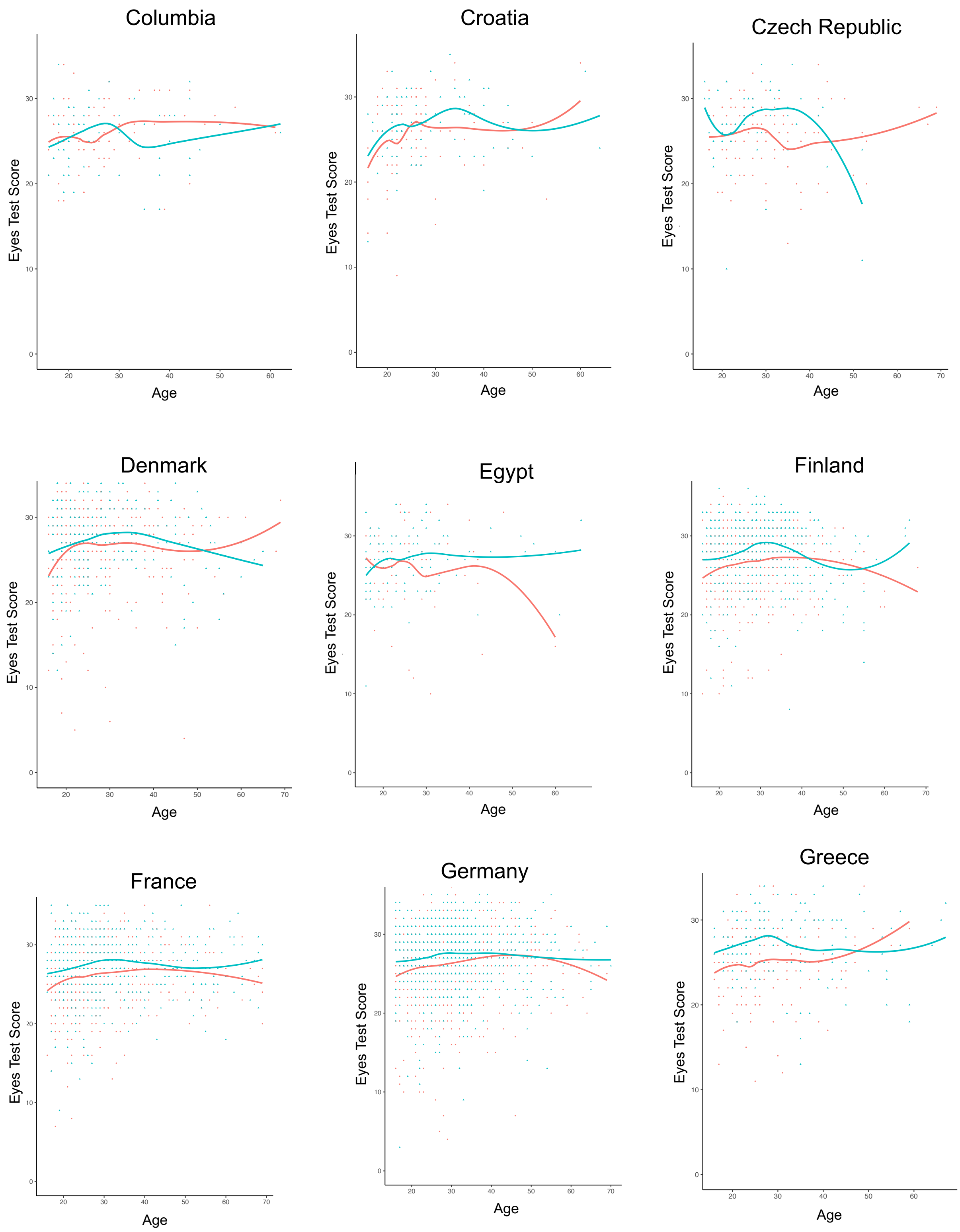
Columbia
Croatia
Czech Republic
Denmark
Egypt
Finland
France
Germany
Greece
Eyes Test Score
Age

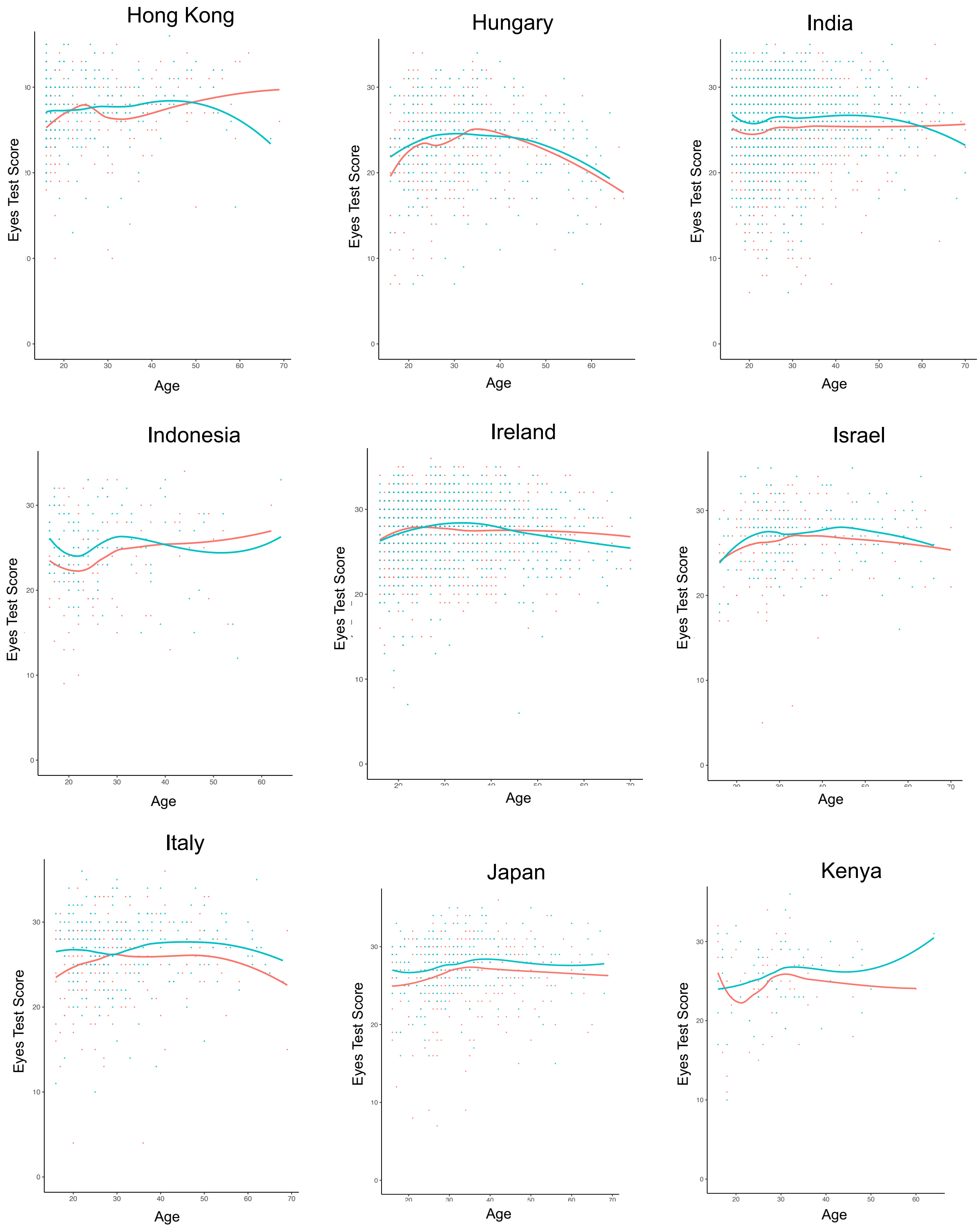
Hong Kong
Hungary
India
Indonesia
Ireland
Israel
Italy
Japan
Kenya
Eyes Test Score
Age

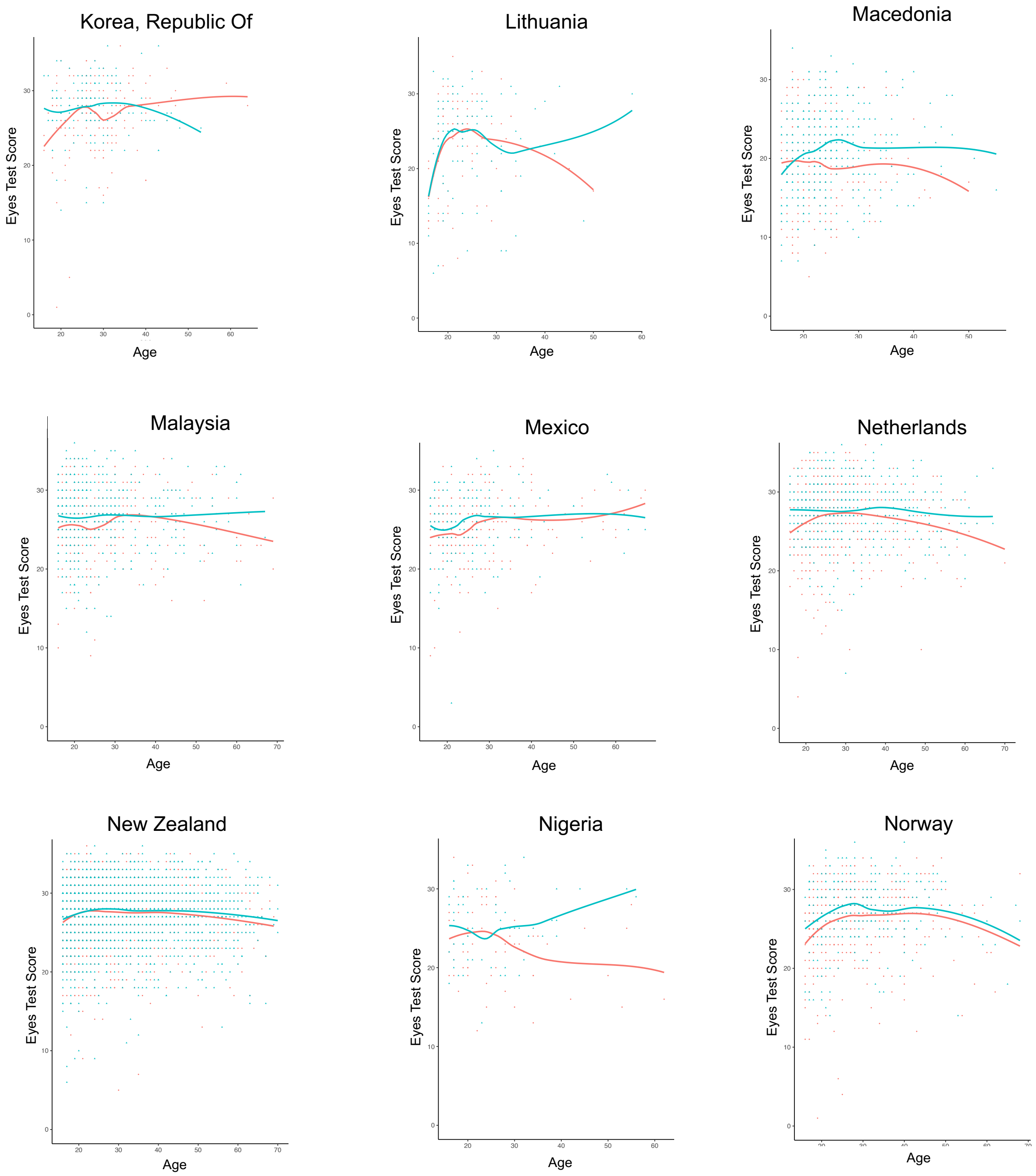
Korea, Republic Of
Eyes Test Score
Age
Lithuania
Eyes Test Score
Age
Macedonia
Eyes Test Score
Age
Malaysia
Eyes Test Score
Age
Mexico
Eyes Test Score
Age
Netherlands
Eyes Test Score
Age
New Zealand
Eyes Test Score
Age
Nigeria
Eyes Test Score
Age
Norway
Eyes Test Score
Age

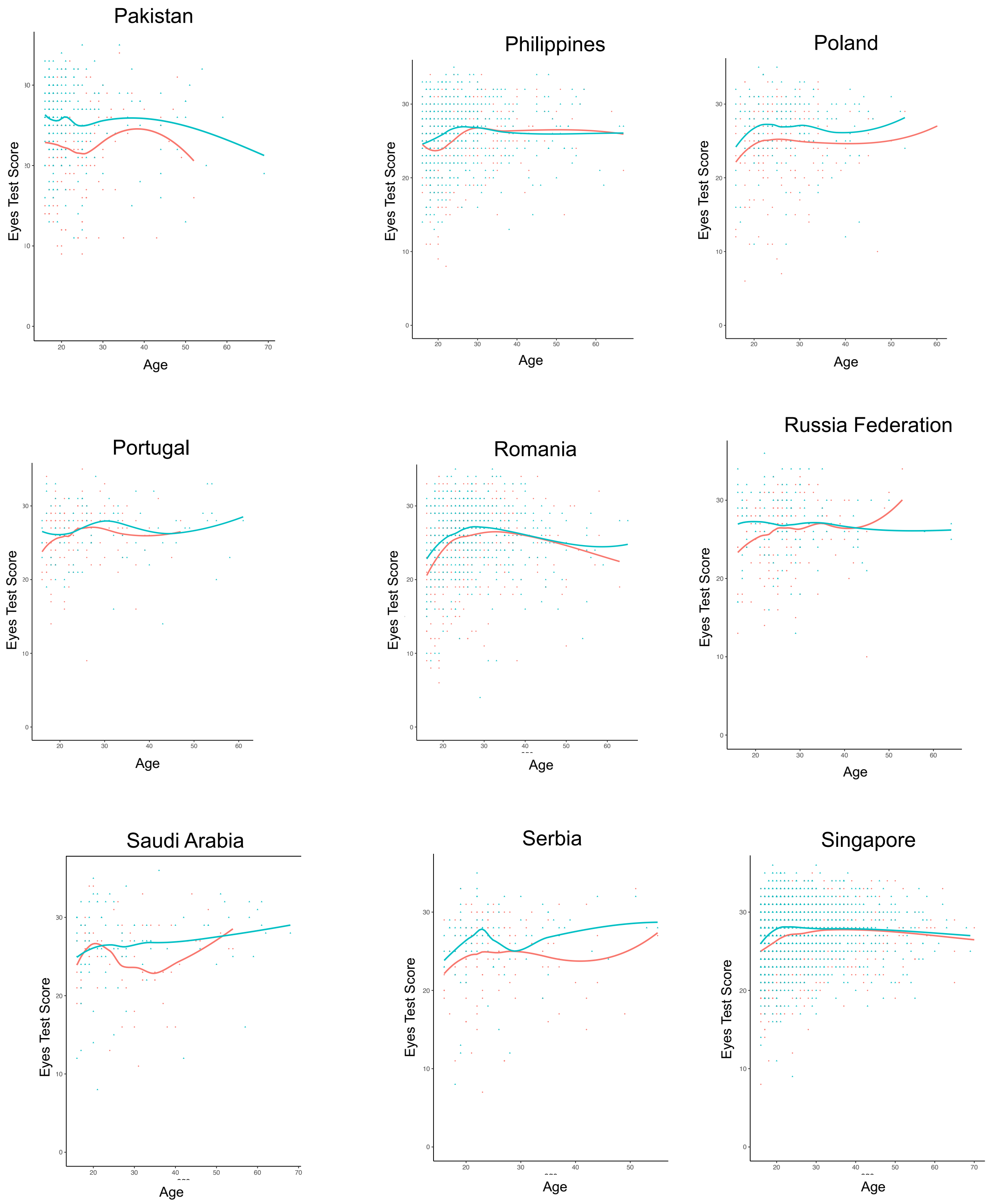
Pakistan
Philippines
Poland
Portugal
Romania
Russia Federation
Saudi Arabia
Serbia
Singapore
Eyes Test Score
Age

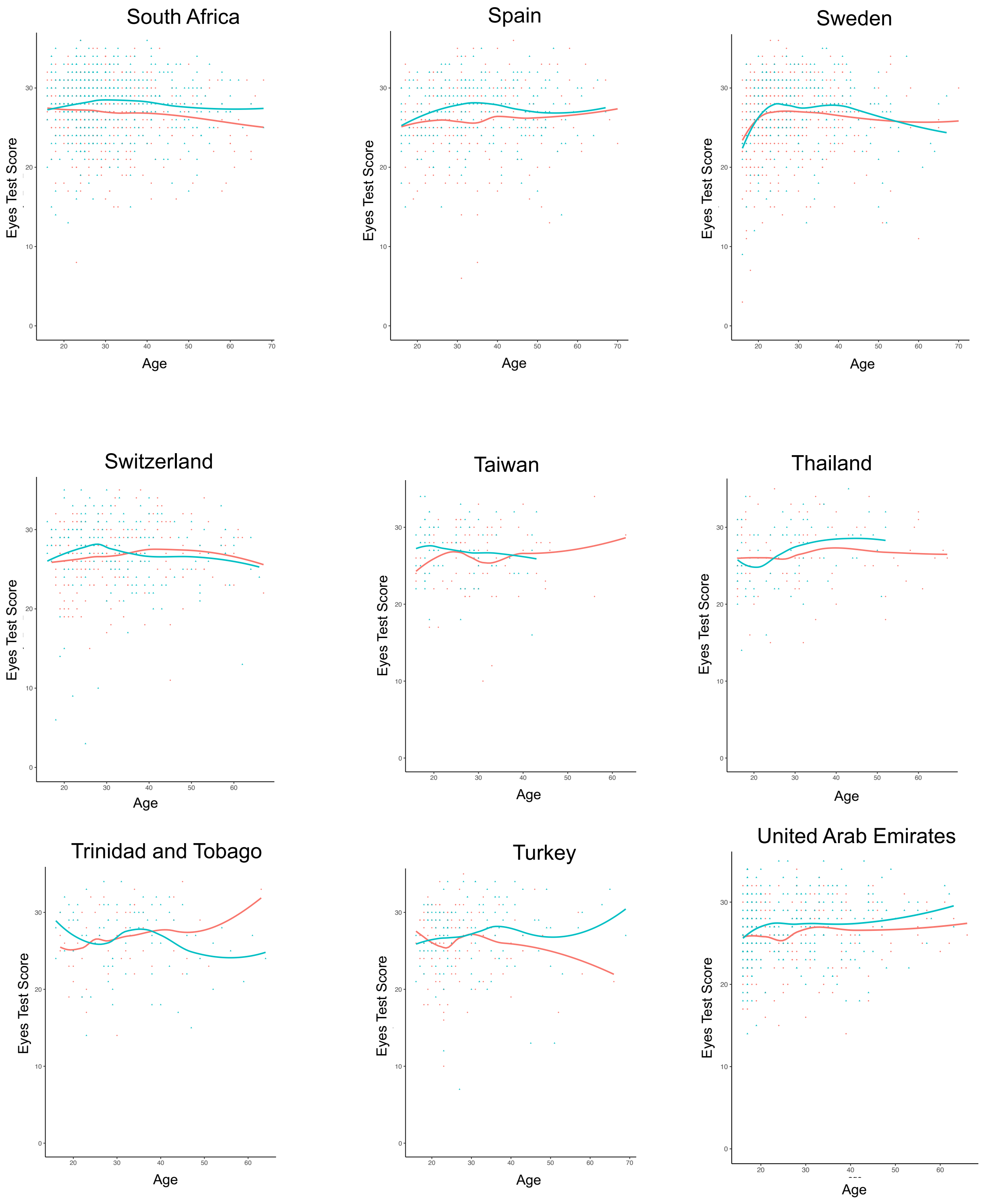
South Africa
Spain
Sweden
Switzerland
Taiwan
Thailand
Trinidad and Tobago
Turkey
United Arab Emirates
Eyes Test Score
Age

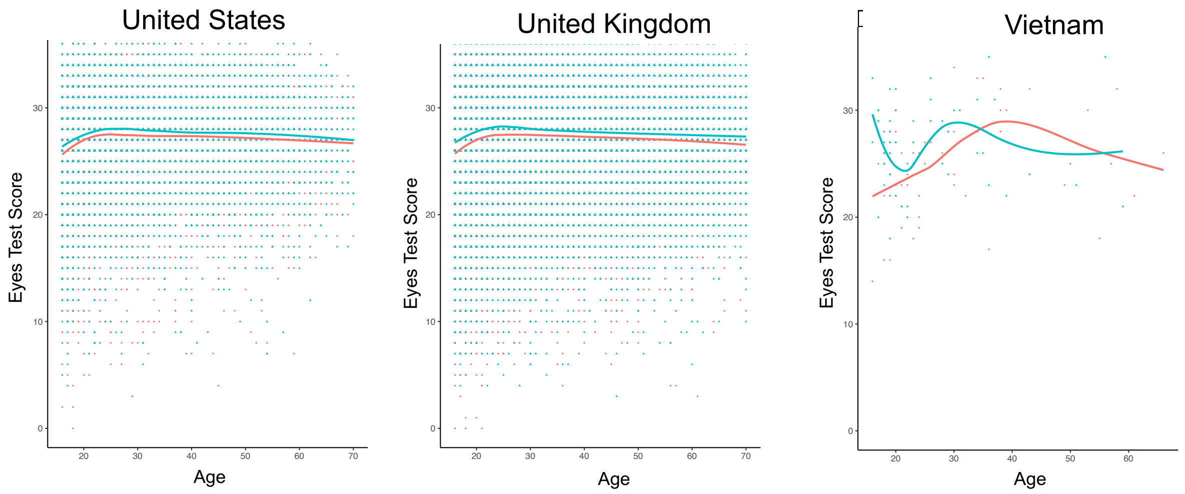

This figure provides facet plots individually by country showing age trends on Eyes Test scores from 16 to 70 in the discovery dataset. Females and their trend line are turquoise-coloured and males and their trend line are salmon-coloured. The results are based on LOESS regression for each country by sex. The y-axis on each plot are Eyes Test scores and the x-axis is age.
